# Decomposing Neural Representational Patterns of Discriminatory and Hedonic Information during Somatosensory Stimulation

**DOI:** 10.1523/ENEURO.0274-22.2022

**Published:** 2023-01-03

**Authors:** James H. Kryklywy, Mana R. Ehlers, Andre O. Beukers, Sarah R. Moore, Rebecca M. Todd, Adam K. Anderson

**Affiliations:** 1 Department of Psychology, Lakehead University, Thunder Bay, Ontario P7B 5E1, Canada; 2 Department of Psychology, The University of British Columbia, Vancouver, British Columbia V6T 1Z4, Canada; 3 University Medical Centre Hamburg-Eppendorf, Hamburg 20251, Germany; ^4^Department of Psychology, Princeton University, Princeton, NJ 08540; 5 Department of Medical Genetics, The University of British Columbia, Vancouver, British Columbia V6H 3N1, Canada; 6 Department of Human Development, Cornell University, Ithaca, NY 14853; 7 Djavad Mowafaghian Centre for Brain Health, The University of British Columbia, Vancouver, British Columbia V6T 1Z3, Canada

**Keywords:** fMRI, hedonic, pattern component modeling, representational similarity, somatosensation

## Abstract

The ability to interrogate specific representations in the brain, determining how, and where, difference sources of information are instantiated can provide invaluable insight into neural functioning. Pattern component modeling (PCM) is a recent analytic technique for human neuroimaging that allows the decomposition of representational patterns in brain into contributing subcomponents. In the current study, we present a novel PCM variant that tracks the contribution of prespecified representational patterns to brain representation across areas, thus allowing hypothesis-guided employment of the technique. We apply this technique to investigate the contributions of hedonic and nonhedonic information to the neural representation of tactile experience. We applied aversive pressure (AP) and appetitive brush (AB) to stimulate distinct peripheral nerve pathways for tactile information (C-/CT-fibers, respectively) while patients underwent functional magnetic resonance imaging (fMRI) scanning. We performed representational similarity analyses (RSAs) with pattern component modeling to dissociate how discriminatory versus hedonic tactile information contributes to population code representations in the human brain. Results demonstrated that information about appetitive and aversive tactile sensation is represented separately from nonhedonic tactile information across cortical structures. This also demonstrates the potential of new hypothesis-guided PCM variants to help delineate how information is instantiated in the brain.

## Significance Statement

This work provides a novel brain imaging analyses that enables the decomposition of brain states into subcomponent states each associated with distinct patterns of experience or information. This technique is applied to human neuroimaging data acquired during hedonic touch to demonstrate a dual role of somatosensation in affective and sensory experience. The analytic advancement highlights exciting new developments in human neuroscience, allowing for the decomposition of human experience into discrete representation state both within and across brain areas.

## Introduction

One of the holy grails of cognitive neuroscience is to be able to “read out” the content of cognition from nothing but patterns of brain activation. Whereas multivariate approaches that emphasize “decoding” are effective at classification or mapping distance in representational space, current efforts are focusing on how to model the specific content being instantiated by patterns of BOLD response across voxels (Kriegeskorte et al., 2008; Kriegeskorte and Kievit, 2013; Visser et al., 2013, 2015, 2016). Here, we present one such approach, focusing on using a variant of pattern component modeling (PCM; Diedrichsen et al., 2011, 2018; Kriegeskorte and Kievit, 2013) that fits combinations of prespecified conceptual similarity patterns to observed neural representational patterns to investigate instantiations of sensory and hedonic tactile information in the CNS.

Sensory experiences, such as the embrace of a loved one or the pain of a stubbed toe, can be broken down into two central components: discrimination of the sensory information and the associated hedonic response. The information processed by sensory systems is typically viewed as objective, forming representations of a tangible external environment that are not yet colored by emotional evaluations. Yet, in the somatosensory system, there is strong evidence that hedonic information (good vs bad) is coded by peripheral afferents before any cortical processing (Iggo, 1959, 1960; Vallbo et al., 1999). This indicates that there are pathways of tactile sensation that signal pleasant and painful emotional content, rather than “pure” sensory experience (Craig, 2015; Kryklywy et al., 2020). Yet while discriminatory and hedonic information are clearly dissociable in the peripheral nervous system (PNS), investigating the dissociability versus integration of these signals as instantiated in the CNS remains a challenge, in part because of the limited analytic techniques for parcellating overlapping patterns of representation in the brain. The current work presents a novel approach to the analysis of human neuroimaging data that decomposes multivariate representational patterns (described via representational similarity analyses; RSAs) into predefined representational patterns of interest (POIs) that model the information content instantiated by the BOLD response. Specifically, in the current study, we interrogated BOLD patterns evoked by aversive pressure (AP) and pleasant caress to examine instantiation of hedonic and discriminative somatosensory information in the CNS.

The somatosensory system contains multiple functional subsystems, with specific peripheral nerves serving as labeled lines for information traveling into the CNS (McGlone and Reilly, 2010; McGlone et al., 2014). Fast large-diameter myelinated afferent fibers (A-fibers) carry signals pertaining to sensory discrimination, while slower unmyelinated fiber pathways [C-fibers, Qiu et al., 2006; C-tactile (CT) fibers, Löken et al., 2009; Marshall et al., 2019] carry signals of the hedonic value of the sensation (pain and pleasure, respectively). A-fibers predominantly convey information about the timing and location of cutaneous sensory stimulation (McGlone and Reilly, 2010), with some fibers specialized for nociception (Nagi et al., 2019). Additional small-diameter unmyelinated afferent pathways support the hedonic response to touch, conveying information about affective aspects of aversive touch and nociception. At the cortical level, primary somatosensory cortex (S1) is the dominant entry point for information carried along myelinated cutaneous pathways. The extent to which hedonic and nonhedonic peripheral pathways remain segregated on their entry into cortical areas, including S1, remains unclear, in part because of an inability to differentiate between multiple distinct representations patterns versus a single integrated representational pattern instantiated in a single brain region.

There is evidence that integration of hedonic information into discriminatory touch representations in these early sensory structures occurs through centrally-mediated appraisal of pain and pleasure (Bushnell et al., 1999; Gazzola et al., 2012). This observation is consistent with conventional views positing that modulation of sensory information by emotion relies on re-entrant projections from higher order structures assessing hedonic value [e.g., prefrontal cortices (PFCs), insula (Ins), amygdala] to the sensory cortices (Pessoa and Adolphs, 2010; Rolls, 2019). Yet, evidence for peripheral labeling of affective information suggests that not all affective modulation of sensory signals is the result of central feedback (Qiu et al., 2006). Considerable evidence exists to support a neural bases of pain-coding in the periphery (McGlone and Reilly, 2010; Nagi et al., 2019). Anatomical projection studies in primates indicate that information carried along unmyelinated C-fiber pathways does not project to the entirety of S1, as observed for A-fibers. Rather, it projects to an anterior region of S1 (insula-adjacent area 3a; Whitsel et al., 2009; Vierck et al., 2013), with additional direct projections to the insula, anterior cingulate cortex (ACC), and PFC (Baumgärtner et al., 2006; Qiu et al., 2006). Similarly, recently identified pathways, C-tactile (CT) fibers, have been shown to carry information about caress or pleasant touch (Löken et al., 2009; Marshall et al., 2019). These fibers originate from mechanoreceptors located in hairy skin rather than the glabrous (i.e., hairless) skin of the palms, where previous research had focused (McGlone and Reilly, 2010; McGlone et al., 2014; Marshall et al., 2019). These CT-fiber afferents respond preferentially to touch that is subjectively perceived as pleasant caress (Olausson et al., 2002; Löken et al., 2009; Croy et al., 2016). Thus, in the cutaneous system there may be distinct parallel representations for tactile stimulation beginning from the point of contact, carried through hedonic labeled lines that independently inform the experience of hedonic value.

Previous functional magnetic resonance imaging (fMRI) studies examining neural substrates of affective-tactile processing have investigated either C- or CT-fiber pathways, but not both. The independent examination of C- and CT-fibers does not allow for the dissociation between these two distinct systems and is unable to discriminate valence-specific hedonic information from general tactile salience and arousal. Moreover, these studies have also relied predominantly on univariate statistical approaches (Olausson et al., 2002; Löken et al., 2009; McGlone et al., 2014). Univariate approaches have limited ability to discriminate specific information that is represented within a region, particularly perceptual and hedonic information represented by different sensory systems (Chikazoe et al., 2014; Todd et al., 2020). By contrast, multivariate analyses, including representational similarity analyses (Kriegeskorte et al., 2008) allow the examination of population-based neuronal coding in a multidimensional representational space. When further analyzed through pattern component modeling (PCM; Kriegeskorte and Kievit, 2013; Diedrichsen et al., 2018), this representational space can be decomposed into weighted subcomponents of experience (Diedrichsen et al., 2018), thus representing neural activity as an integration of multiple heterogeneous sets of overlapping neural representations. In the present study, we implemented an innovative analytic approach that uses theory-guided components to perform PCM. We generated combinations of predefined similarity matrices in an approach that is a variation of previously described applications of a priori conceptual models of representational dissimilarity matrices (mRDM; Kriegeskorte et al., 2008; Kriegeskorte and Kievit, 2013; Popal et al., 2019). We refer to our hypothesized similarity models as patterns of interest (POIs), each of which characterizes a correlation pattern that would be expected given perfect representation of a single vector of information, e.g., painful touch. Whereas previous approaches fit single hypothesized conceptual similarity/dissimilarity matrices to the observed data, the current POI (Kryklywy et al., 2021) looks for the simultaneous best fit of combinations of multiple models to observed representational patterns extracted from predefined regions of interest (ROIs).

**Figure 1.**
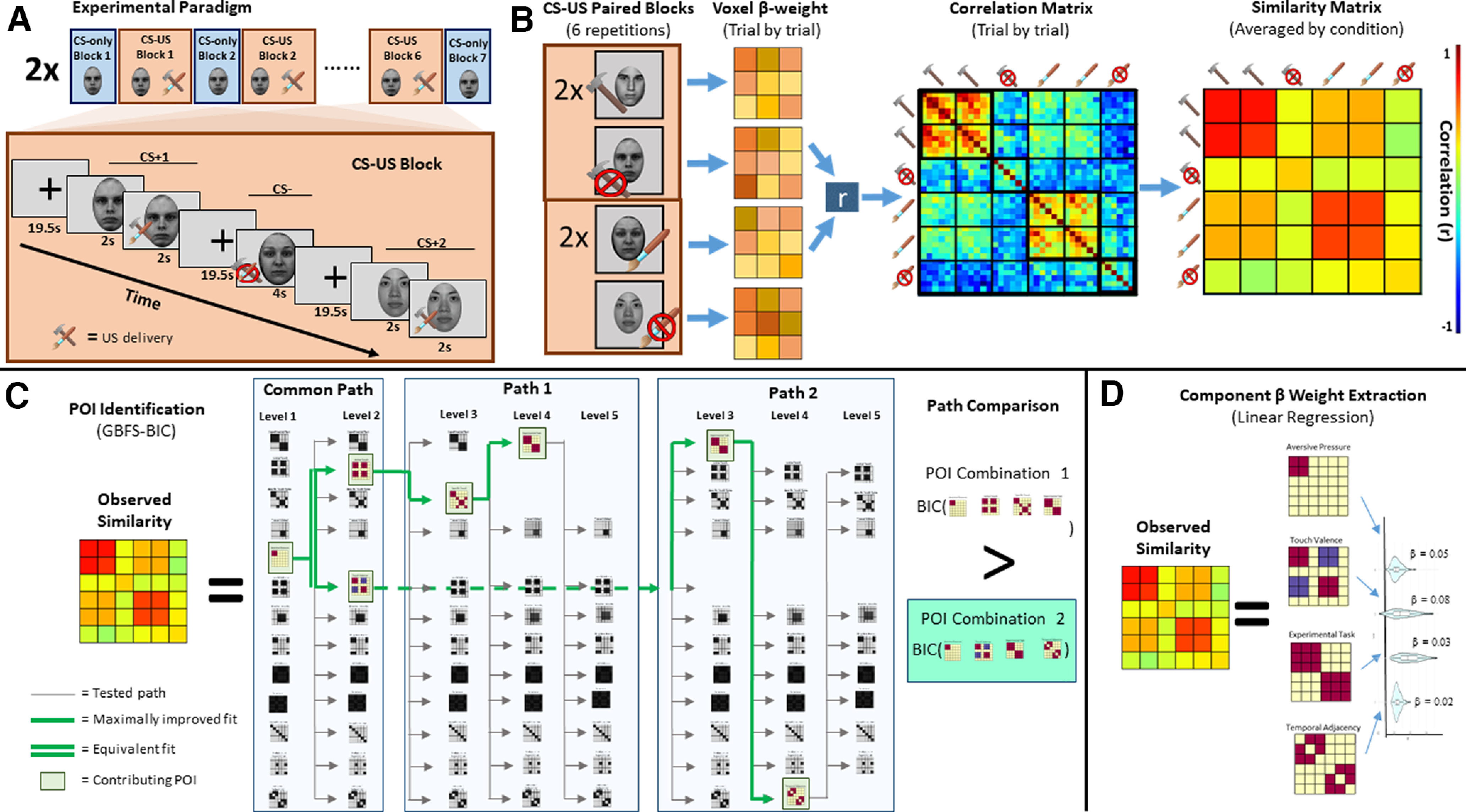
***A***, Experimental time course. Participants completed tactile visual conditioning tasks. Only data collected during CS-US paired blocked will be presented. ***B***, Representational similarity analyses (RSA). RSA was conducted correlating all experimental trials independently. Resultant Pearson correlation coefficients were averaged across conditions (removing autocorrelation) to create a 6 × 6 condition similarity matrix comparing all conditions of interest. ***C***, Pattern of interest (POI) identification. A greedy best-first search algorithm (GBFS) with Bayesian information criterion (BIC) was used to identify the combination of POIs that best predicted experimental data. Level-1 search independently fit 13 unique POIs to observed representation similarity. The POI with optimal fit was combined with all remaining models (Level 2) to determine the combination of POI represented in the ROI. This process was done iteratively until the addition of none of the POIs improved model fit. ***D***, POI weighting. Multiple regression allowed extraction of β value indicating the representational strength for each contributing POI.

Functional neuroimaging data were collected while participants received aversive pressure on the thumb or appetitive caress stimulation on the forearm (Fig. 1*A*) and viewed images of faces with neutral expressions. RSA conducted on the BOLD signal identified similarity/dissimilarity between tactile conditions that not only represent distinct discriminatory patterns but also putatively stimulate distinct fiber pathways for aversive and appetitive tactile experience (Fig. 1*B*). POIs were created for task-relevant information content and included specific aspects of tactile and emotional experience. Bayesian information criterion (BIC) analyses were then performed to characterize the combination of POIs that best predicted observed similarity patterns of neural activity in each ROI. This allowed us to identify and weigh dissociable representations of discriminative and hedonic tactile signals, revealing potential C- and CT-fiber pathways projection. Critically, these analyses allow us to fit multiple overlapping pattern components simultaneously and identify the best fitting combination, rather than fitting each component alone and comparing the representational strength of each component in isolation. We believe that this feature affords a nuanced and faithful glimpse into how a brain region may hold and process multiple sources of information.

We predicted that modeled representations of the hedonic components of the tactile stimulation in frontotemporal cortices, including vmPFC, ACC, and insula would be distinct from representations in primary somatosensory cortices. This would demonstrate the dual coding of somatosensation. We expected that dissociable representational patterns for appetitive versus aversive tactile stimulation would be identified in the insula and vmPFC, as these regions may receive direct unprocessed information from hedonic-labeled lines. Patterns observed in the ACC were predicted to be most heavily weighted toward representation of aversive touch, consistent with this region’s preferential activation in response to modality-general pain.

## Materials and Methods

### Participants

A total of 121 participants ( $\bar{x}$ _age_ = 21.1, SD = 2.8; 41F) were recruited from a pool of 488 participants at Cornell University who had completed a large-scale behavioral study examining individual differences in responsiveness to reward and punishment (Moore, 2017). Of the data collected for the present study, we were unable to complete preprocessing for 54 participants. Multiecho (ME) preprocessing (described below) failed to generate the required denoised datasets (i.e., *medn* files) for 25 participants, while we were unable to obtain convergence in the independent component analyses (ICA) for an additional 14 participants. Data files (e.g., imaging run, stimulus onset timings, motion correction files) were missing for 12 participants. Two participants were excluded because of motion artifacts and a final participant was excluded to nonstandardized data collection. Results from the remaining 67 participants are reported. All participants gave written, informed consent and had normal or corrected-to-normal vision. Participants were prescreened for a history of anxiety and depression as well as other psychopathology, epilepsy, and brain surgery. Prescreening was followed up in person by an additional interview to ensure inclusion criteria were met. As this study was conducted as part of larger research program, all participants provided saliva samples for genotyping, and fecal sample for microbiome analyses. The experiment was performed in accordance with the Institutional Review Board for Human Participants at Cornell University.

### Stimuli and apparatus

Photographs of three male and three female faces with neutral expressions were chosen from the Karolinska directed emotional faces picture set (Goeleven et al., 2008). These face images were used as conditioned stimuli (CS) in two classical conditioning paradigms, one aversive and one appetitive. Analysis here focuses only on the trials that included tactile stimulation = paired with the faces, as effects of conditioning are reported elsewhere (Ehlers et al., 2021). Unconditioned stimuli (US) consisted of either aversive pressure delivered to the right thumb, or appetitive caress to the participant’s left forearm. These tactile manipulations were aimed to maximally activate C- and C-tactile somatosensory afferents, respectively. Aversive pressure stimuli were delivered using a custom designed hydraulic device (Giesecke et al., 2004; López-Solà et al., 2010) capable of transmitting controlled pressure to 1-cm^2^ surface placed on the subjects’ right thumbnail. Applied pressure levels were individually calibrated for each participant before the experiment to ensure that the pressure intensity was experienced as aversive but not excessively painful. Light appetitive caresses lasting ∼4 s were manually applied to the left forearm with a brush by a trained experimenter to maximally activate CT-fiber pathways (McGlone et al., 2014). Only participants who had previously demonstrated reliable affective responses to the tactile manipulations in the larger behavioral study (e.g., positive responses to caress; see below for details) were invited to participant in the scanning session, as this indicated likely recruitment of C- and CT-fiber pathways of the hedonic tactile manipulations (which can be subject to interparticipant variability).

### Procedure

While undergoing functional MR scanning, participants completed two separate conditioning tasks (appetitive conditioning and aversive conditioning), each involving a series of tactile and visual pairings (Fig. 1*A*; Visser et al., 2015). In each task, participants completed seven CS-only blocks interleaved with six CS-US paired blocks. Single blocks of either the CS-only or the CS-US pairing contained one presentation of each facial stimulus (i.e., three face stimuli, two CS+ and one CS−, per block of each conditioning task). Individual trials consisted of an initial fixation period (19,500 ms) followed by the presentation of a face (4000 ms). A fixed and long interstimulus interval (19,500 ms) was included in the experimental design to reduce intrinsic noise correlations and enable trial by trial analyses by means of RSA (Visser et al., 2013, 2016). During CS-only trials, all faces were presented without tactile stimulation. During CS-US paired trials, two of three facial stimuli presentations overlapped with tactile stimulation, thus creating two CS+ and one CS−. The US was delivered from the midpoint of the face presentation (2000 ms postonset), remained for the rest of the time the face was visible (2000 ms) and persisted following the offset (2000 ms; total US = 4000 ms). The order of face presentation was randomized within each CS-US paired block. Participants completed two experimental tasks (one for each US, order counterbalanced across participants), totaling 26 blocks (six CS-US paired and seven CS only blocks for each US type).

Note that this paradigm was designed to target two distinct questions: (1) How and where are discriminatory and hedonic tactile signals represented in the brain? and (2) How do novel affective associations change the neural representation of conditioned stimuli? The current work focuses on the former question. Importantly, trials described as CS− during CS-US pairings are not independent of tactile stimulation. Rather, these trials lacked pain or brush stimulation, but participants still experienced tactile stimulation from the scanner, their clothes etc.

### MRI acquisition and preprocessing

MR scanning was conducted on a 3 Tesla GE Discovery MR scanner using a 32-channel head coil. For each subject, a T1-weighted MPRAGE sequence was used to obtain high-resolution anatomic images [TR = 7 ms, TE = 3.42 ms, field of view (FOV) 256 × 256 mm, slice thickness 1 mm, 176 slices]. Functional tasks were acquired with the following multiecho (ME) EPI sequence: TR = 2000 ms, TE1 = 11.7 ms, TE2 = 24.2 ms and TE3 = 37.1 ms, flip angle 77°; FOV 240 × 240 mm. These parameters are consistent with recent work demonstrating improved effect-size estimation and statistical power for multiecho acquisition parameters (Lombardo et al., 2016). Specifically, the multiecho sequence was chosen because of its enhanced capacity for differentiating BOLD and non-BOLD signal (Kundu et al., 2012, 2014), as well as its sensitivity for discrimination of small nuclei in areas susceptible to high signal dropout (Markello et al., 2018). A total of 468 volumes (102 slices, thickness 3.5 mm; 72 × 72 acquisition matrix, 3.33 × 3.33 mm) was acquired for each functional run. Pulse and respiration data were acquired with scanner-integrated devices.

Preprocessing and analysis of the fMRI data were conducted using Analysis of Functional NeuroImages software (AFNI; Cox, 1996) and the associated toolbox *meica.py* (Kundu et al., 2014, 2017). For maximal sensitivity during multivariate pattern detection, no spatial smoothing was performed on the data (Haynes, 2015). Preprocessing of multiecho imaging data followed the procedural steps outlined by Kundu and colleagues (Kundu et al., 2012, 2013), as described below, and used independent component analyses to define a set of components using their TE dependence to be individually classified as BOLD or non-BOLD (e.g., motion artifact). An optimally combined (OC) dataset was generated from the functional multiecho data by taking a weighted summation of the three echoes, using an exponential T2* weighting approach (Posse et al., 1999). Multiecho principal components analysis (PCA) was first applied to the OC dataset to reduce the data dimensionality. Spatial independent components analysis (ICA) was then applied and the independent component time-series were fit to the preprocessed time-series from each of the three echoes to generate ICA weights for each echo. These weights were subsequently fitted to the linear TE-dependence and TE-independence models to generate F-statistics and component-level κ and ρ values, which, respectively, indicate BOLD and non-BOLD weightings. The κ and ρ metrics were then used to identify non-BOLD-like components to be regressed out of the OC dataset as noise regressors. Regressor files of interest were generated for all individual trials across the experiment, modeling the time course of each stimulus presentation during each run (36 total events: two tasks × six CS-US blocks × three CS, with each event beginning at the face presentation onset). The relevant hemodynamic response function was fit to each regressor for linear regression modeling. This resulted in a β coefficient and t value for each voxel and regressor. To facilitate group analysis, each individual’s data were transformed into the standard brain space of the Montreal Neurologic Institute (MNI).

### fMRI analyses: structural regions of interest

To assess tactile [pressure and caress and nonspecific touch (nST)] and hedonic (pressure vs caress) representations in neural patterns, nine bilateral regions of interest (ROIs) were generated from the standard anatomic atlas (MNIa_caez_ml_18) implemented with AFNI. Sensory ROIs included primary somatosensory cortex (S1), secondary somatosensory cortex (S2), primary visual cortex (V1) and ventral visual structures (VVS) while integrative ROIs included amygdalae, ventromedial prefrontal cortex (vmPFC; Posse et al., 1999), anterior cingulate cortex (ACC), and both the anterior and posterior divisions or insular cortex (Ins; for review of the functional and histologic divisions of this region, see Nieuwenhuys, 2012). S1 and V1 were selected as the primary sites of tactile and visual information, respectively. VVS were chosen because of their role in visual classification (Kanwisher et al., 1997; Kravitz et al., 2013). Amygdala, vmPFC, ACC and posterior/anterior Ins divisions were selected for their hypothesized roles in affect and pain representations subdivisions (Anderson and Phelps, 2002; for rationale behind multiple insular ROIs, see Cauda et al., 2012; Chikazoe et al., 2014; Orenius et al., 2017; Kragel et al., 2018). For extended details on defining our ROI, see Table 1.

**Table 1:** Regions of interest

Region	Abbreviation	Traditional processing	Volume (mm^3^)	Atlas*	Label**
Primary somatosensory cortex	S1	Sensory	60,200	MNIa_caez_ml_18:	Postcentral gyrus
Left S1	lS1	Sensory		MNIa_caez_ml_18:	Left postcentral gyrus
Right S1	rS1	Sensory		MNIa_caez_ml_18:	Right postcentral gyrus
Secondary somatosensory cortex	S2	Sensory	12,981	MNIa_caez_ml_18:	L/R rolandic operculum (posterior to the anterior commissure; y > 0)
Primary/secondary visual cortex	V1	Sensory	76,968	MNIa_caez_ml_18:	Lingual gyrus + calcarine gyrus + cuneus (y > 60)
Ventral visual structures	VVS	Sensory	105 664	MNIa_caez_ml_18:	Inferior temporal gyrus + inferior occipital gyrus + fusiform gyrus
Amygdalae	Amy	Affect	3664	MNIa_caez_ml_18:	Amygdala
Ventromedial prefrontal cortex	vmPFC	Affect Cognitive	42,160	MNI_vmPFC:	Entire atlas
Anterior cingulate cortex	ACC	Sensory Affect Cognitive	26,528	MNIa_caez_ml_18*^a^*^:^ MNI_vmPFC*^b^*^:^	[anterior cingulate + middle cingulate]*^a^* minus overlap*^b^* (anterior to the anterior commissure; y < 0)
Insula (anterior)	aIns	Affect Interoceptive	17,728	MNIa_caez_ml_18:	Insula lobe (anterior to the anterior commissure; y < 0)
Insula (posterior)	pIns	Sensory Affect	8296	MNIa_caez_ml_18:	Insula lobe (posterior to the anterior commissure; y > 0)

*All atlases were transformed to MNI space before their implementation or manipulation.

**All labels refer to the bilateral structures, unless otherwise indicated.

### fMRI analyses: RSA

In order to identify and compare representational patterns elicited by the experimental conditions, representational similarity analysis (RSA; Mur et al., 2009; Kriegeskorte and Kievit, 2013; Fig. 1*B*) was performed using the PyMVPA Python package (Hanke et al., 2009). For each participant, a vector was created containing the spatial patterns derived from β coefficients from each voxel related to each face-touch pairing in each ROI. Pairwise Pearson coefficients were calculated between all vectors of a single ROI, thus resulting in a similarity matrix containing correlations between patterns of β weights for all trials for each participant (i.e., how closely the pattern of voxel activation elicited in one trial resembles the patterns of voxel activation observed in all other trials). Fisher transformations were performed on all similarity matrices to allow comparisons between participants. Following the identification of trial-by-trial correlations, each matrix weas down-sampled to represent the condition-by-condition similarity. Thus, each cell in the presented similarity matrices is the average value of all correlations between β-values elicited during the relevant trial types. The average correlation within each condition (i.e., correlation between all repetitions of a single face-touch pairing) is the mean of 30 individual correlations (6 × 6 trial matrix trials with auto-correlations removed), while the average correlation between conditions (i.e., correlations between two different face-touch pairings) is the mean of 36 individual correlations (complete 6 × 6 trial matrix). Correlation matrix transformations were performed using MATLAB (The MathWorks).

### fMRI analyses: patterns of interest

Pattern component modeling is a method to decompose multivariate similarity patterns into distinct subcomponents (Kriegeskorte et al., 2008; Diedrichsen et al., 2011, 2018; Kriegeskorte and Kievit, 2013). Here, we developed a novel theory-guided implementation of PCM that determines the contribution of known sources of information to overall representational patterns observed in the brain. Patterns of interest (POIs) were created illustrating the similarity matrices that would be observed if the data were to ideally represent a single type of information perfectly. POIs are functionally similar to conceptual dissimilarity models (mRDM) for modeling content of RSA patterns (Kriegeskorte et al., 2008; Kriegeskorte and Kievit, 2013; Popal et al., 2019) in that they act to fit observed data to theory-based prespecified representational patterns. Critically, however, the current approach is distinct from these in that it acts to fit combinations of conceptual patterns of interest to a given brain region, rather than individual models.

Thirteen POIs were designed to represent unique task-relevant representational patterns could be expected to be elicited during the current task. For example, one POI would illustrate what the data pattern would look like if the voxels across a given ROI were to represent only the positive or negative valence of the touch as opposite poles of a bipolar scale. Note that the POIs were designed to capture information about the specific manipulations of the current experiment as well as cognitive processes associated with the ROIs defined in this study. The POIs we described have a particular focus on potential representations of tactile experience. Thus, they may not accurately reflect information represented in brain regions processing predominantly nontactile information and are not intended to be an exhaustive model of all potential representational states. The following is an extended description of each POI, with additional description and visualization presented in Table 2.

**Table 2:** POI glossary

Name	Abbreviation	Description	Visual representation*
Experimental task	ET	*r* = 1 for all comparisons between all trials in each conditioning task. • i.e., within separate aversive and appetitive tasks, all the trials (in the presence and absence of touch) are represented as similar.	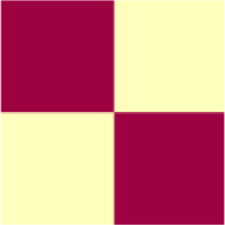
Nonspecific touch	nST	*r* = 1 for all comparisons between all trials where a tactile manipulation occurred. • i.e., shared representation both within and between aversive pressure and appetitive caress trials.	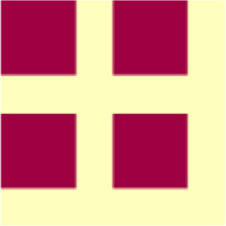
Specific touch	ST	*r* = 1 for all comparisons between all trials where an identical tactile experience occurred. • i.e., shared representation within, but not between trials with aversive pressure, trials with appetitive caress and unpaired trials, respectively.	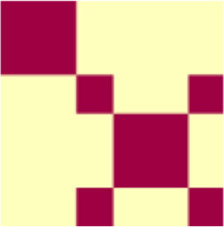
Appetitive brush	AC	*r* = 1 for all comparisons between all trials that involved the delivery of an appetitive caress to the participant’s arm.	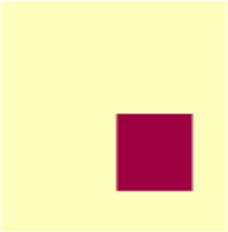
Aversive pressure	AP	*r* = 1 for all comparisons between all trials that involved the delivery of aversive pressure to the participant’s thumb.	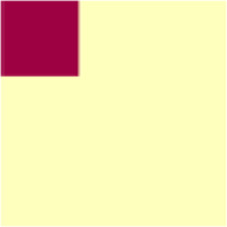
Touch valence	TV	*r* = 1 for all comparisons between trials involving tactile stimulation within each conditioning task. *r* = −1 for all comparisons between trials involving tactile stimulation between conditioning tasks. NOTE: This reflects a linear representation of hedonic *tactile* information.	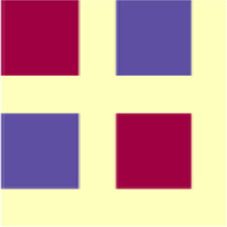
Positive events	PE	*r* = 1 for all comparisons between all trials experienced as positively valenced relative to its experimental task. • i.e., tactile stimulation trials in the appetitive task are represented as similar to trials with no stimulation in the aversive task.	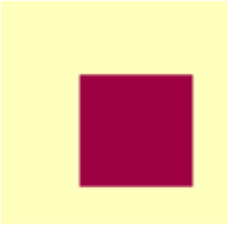
Negative events	NE	*r* = 1 for all comparisons between all trials experienced as negatively valenced relative to its experimental task. • i.e., tactile stimulation trials in the aversive task are represented as similar to trials with no stimulation in the appetitive task.	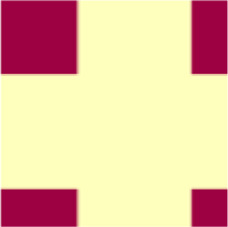
All valence	AV	*r* = 1 for all comparisons between trials containing positive events and between trials containing negative events. *r* = −1 for all comparisons between trials of positive events and negative events. *NOTE: This reflects a linear representation of *all* hedonic information.	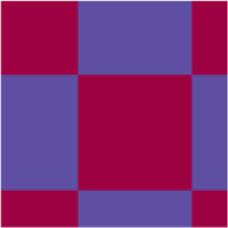
Salience	Sa	*r* = 1 for all comparisons between all trial with highly tactile salience. • shared representation both within and between trials with appetitive and aversive stimulation.	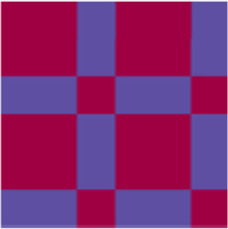
		*r* = 1 for all comparisons between all trial with minimal tactile salience. • shared representation both within and between trials with no appetitive/aversive stimulation.	
		*r* = −1 for all correlation between highly and minimally salient trials.	
Facial stimulus	FS	*r* = 1 for all comparisons between trials where the visual stimulus presented (i.e., the face) was identical. • i.e., distinct representation for each of the 6 faces (3 faces × 2 tasks).	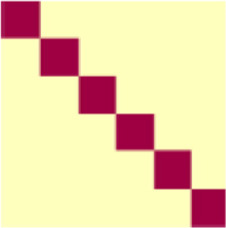
Violation of expectation	VE	*r* = 1 for all comparisons correlation between all trials involving the less probable outcome. NOTE: As there were two faces paired with tactile stimulation compared with one face never paired with tactile stimulation for each experimental task, the less probable outcome was always the unpaired trials.	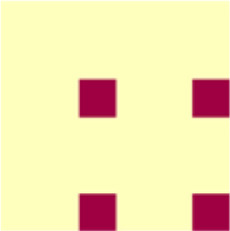
Temporal adjacency	TA	*r* = 1 for all comparisons between all comparisons that included trials that were temporally contained within the same block (i.e., temporally adjacent exposures). NOTE: Because of the removal of autocorrelation from within condition averaging, these did not contain temporally adjacent trials.	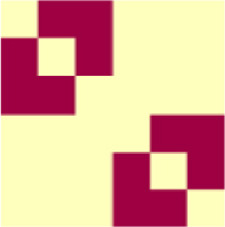

*General matrix structure can be found in Figure 1*B*; red represents *r* = 1, blue represents *r* = −1 and yellow represents *r* = 0.

#### Experimental task (ET)

Experimental task was conceptualized as representational overlap for all trials contained in one conditioning task (i.e., the aversive vs appetitive task). This included both trials with and without tactile stimulation. Ideal representation of Experimental task was defined as perfect correlation (*r* = 1) between all trials in each specific conditioning task. All correlations for trial across conditioning tasks were set to *r* = 0.

#### Nonspecific touch (nST)

Nonspecific touch was conceptualized as identical representations for all trials where a tactile manipulation occurred. Importantly, this POI does not represent any discriminable information between the two tactile manipulations (aversive vs appetitive), but rather defines them as sharing representational space. This POI may be derived from information carried by nonhedonic (A-fiber) or hedonic (C/C-tactile) peripheral channels; nonhedonic signals may indicate quality/strength of tactile information while hedonic signals may indicate emotional saliency of tactile experience. Note, however, that this pattern is unlikely to occur as a result of first order hedonic projection (which are separated into unique positive and negative valence signals), as it includes, by definition, a shared representational space across conditions.

Ideal representation of nonspecific touch was modeled as perfect representational overlap (*r* = 1) between each appetitive brush (AB)-paired trial and all other appetitive brush (AB-AB) trials, each aversive pressure-paired trial and other aversive-pressure (AP-AP) trials, as well as aversive pressure and appetitive brush (AP-AB) trials. All correlations involving no tactile manipulations were defined as *r* = 0.

#### Specific touch (ST)

Specific touch was conceptualized as representing each tactile experience as occupying a unique representation space. Critically, in addition to a unique representational space for appetitive and aversive stimulation, this POI also defines the tactile experience when there was active manipulation as occupying its own representation space. Thus, a pattern similar to this POI cannot be derived from hedonic tactile channels, but rather must rely on alternate signals of discriminative touch (i.e., information carried by A-fibers). Ideal representation of specific touch entailed perfect correlation (*r* = 1) between all trials in which similar tactile experience occurred. This included AP-AP trials, AB-AB trials, and no manipulation (i.e., generic scanner sensation) trials. All additional correlations crossing tactile experience types were set to *r* = 0.

#### Appetitive brush (AB)

Appetitive brush was conceptualized as representing information carried by C-tactile fiber pathways and the associated experience of pleasant touch. Ideal representation of appetitive brush was defined as perfect correlation (*r* = 1) between all trials that involved the delivery of an appetitive brush stroke to the participant’s arm (i.e., AB-AB trials). It should be noted that similarity between these trials could be ascribed to either the tactile sensation of brushing or the hedonic nature of the experience. All additional correlations were set to *r* = 0.

#### Aversive pressure (AP)

Aversive pressure was conceptualized as representing information carried by C-fiber nociceptive pathways and the associated experience of painful stimulation. Ideal representation of aversive pressure was defined as perfect correlation (*r* = 1) between all trials that involved the delivery of aversive pressure to the thumbnail (i.e., AP-AP trials). As with Appetitive brush, it should be noted that similarity between these trials could ascribed to either the tactile sensation of pressure or the hedonic nature of the experience. All additional correlations were set to *r* = 0.

#### Touch valence (TV)

Touch valence represents painful and pleasant tactile experience as opposite ends of a shared linear representational space. Ideal representation of touch valence was defined as a linear representation of the hedonic aspect of the tactile manipulation. For this, all correlations between all AB-AB trials and all AP-AP trials were set to *r* = 1. To represent the contrasting hedonic experience of AB-AP trials, these correlations were set to *r* = −1. All additional correlations were set to *r* = 0.

#### Positive events (PE)

Positive events were conceptualized as a general representation for individual trials that are positively valenced relative to the context in which they are situated. This includes both the positive valence related to the presence of pleasant brushing during the appetitive conditioning task and the absence of painful pressure during the aversive conditioning task. Ideal representation of positive events was defined as perfect correlation (*r* = 1) between all trials where the event was positive relative to its context. Positive trials (PT) included the delivery of a brush stroke during the appetitive conditioning task as well as the absence of pressure in the aversive conditioning task. All additional correlations were set to *r* = 0.

#### Negative events (NE)

Negative events were conceptualized as a general representation for individual trials that negatively valenced relative to the context in which they are situated. This includes both the feeling of painful pressure during the aversive conditioning task, as well as the absence of pleasurable brushing during the appetitive conditioning task. Ideal representation of negative events was defined as perfect correlation (*r* = 1) between all trials where the event was negative relative to the rest of the conditioning task. Negative trials (NT) included the delivery of pressure during the aversive conditioning task as well as the absence of a brush stroke in the appetitive conditioning task. All additional correlations were set to *r* = 0.

#### All valence (AV)

All valence was conceptualized as a linear representation of the hedonic aspects of the experimental procedure relative to the task in which they were situated. Here, “positive events” and “negative events” are independent POIs that are considered to be opposite ends of a shared linear representational space. To represent this, all PT-PT and NT-NT correlations were set to *r* = 1. All PT-NT correlations were set to *r* = −1.

#### Salience (Sa)

Salience was conceptualized as a linear representation of the salience of individual trials, with “high salience” and “low salience” considered as opposite ends of a shared linear representational space. For this, correlations between all highly salient trials (i.e., AB and AP trials) were set to *r* = 1. In addition, correlations between all minimally salient trials (i.e., CS− trials) were also set to *r* = 1. All correlations between highly and minimally salient trials were set to *r* = −1.

#### Face stimulus (FS)

Face stimulus was conceptualized as a unique representational space for each independent facial identity. To model this, FS defines correlations for all trials contained an identical visual CS (six in total, three/experimental task) as *r* = 1. All additional correlations between different CS were set to *r* = 0.

#### Violation of expectation (VE)

Violation of expectation was conceptualized as a shared representation space for the less likely tactile outcome in each conditioning task As there were two CS-US paired trials for each CS-only trials for each experimental task, the less probable outcome was always the CS-only trials. As such, the VE POI modeled all CS-minus-CS-minus correlations (within and across experimental task) as *r* = 1. All additional correlations were set to *r* = 0.

#### Temporal adjacency (TA)

Temporal adjacency was conceptualized as representing any task-irrelevant cognitive processes that may extend beyond single modeled events, and thus inform the representational pattern of temporally adjacent trials. Ideal representation of temporal adjacency was defined as perfect correlation (*r* = 1) between all comparisons that included trials that were temporally contained within the same block (i.e., within each group of three CS presentations). Because of the removal of autocorrelation from within-condition averaging, these did not contain temporally adjacent trials, nor did any correlation of trial between experimental tasks. Thus, correlations for both of these comparisons were set to *r* = 0.

In consideration of the dominant contralateral input to S1 and the lateralized tactile stimulation across tasks (aversive pressure applied to the RIGHT thumbnail, and appetitive caress with a brush applied to the LEFT forearm), two additional POIs were described to facilitate unilateral investigation of this region. There were as follows.

#### Right specific touch (rST)

This POI represents the discriminable tactile experience expected elicited from the right side of the body. It represents both aversive pressure pain (applied to the right thumb) and generic scanner touch (including that occurring on the right side during appetitive brush trials) as dissociable tactile experiences. This POI is predicted to manifest in representation in left S1.

#### Left specific touch (lST)

This POI represents the discriminable tactile experience expected elicited from the left side of the body. It represents both appetitive brushing (applied to the left forearm) and generic scanner touch (including that occurring on the left side during the application of aversive pressure) as dissociable tactile experiences. This POI is predicted to manifest in representation in right S1.

### fMRI analyses: patterns component modeling

To determine the POI combinations that best explained the observed data patterns for each ROI, we conducted Monte Carlo cross-validated (CV) PCM using Bayesian information criterion (BIC) to fit our pattern component models. An uninformed greedy best-first search (GBFS) algorithm (Doran and Michie, 1966) was implemented to identify the best fitting POI combination in a step wise manner (see Fig. 1*C*). This allows us to distinguish between subtle nuances in representational patterns by iteratively layering POIs in a step-wise manner rather than all at once, thus comparing the fit of overtly similar component patterns both independent from, and in combination with, each other. Initial model testing was conducted by fitting each independent POI to the observed similarity between conditions for a given ROI (Level 1). Upon identification of the best fitting POI (POI_B1_), model fitting was conducted on each remaining POI in combination with POI_B_ (Level 2)_._ The POI combination (i.e., POI_B1_ + POI_B2_) that provided the best fit to the ROI data would be held as a constant for model fitting in Level 3. This process was repeated iteratively until no addition of remaining POIs led to an improved fit to the ROI. A ΔBIC > 2 was defined as indicative of an improved fit (Fabozzi, 2014). Following similar equivalency criteria, all POI combinations at a given search level with ΔBIC scores < 2 to the best fitting combination were also extended to path completion (Fabozzi, 2014). This approach allowed for the decomposition of observed representational patterns into multiple unique contributing sources of information. The independent pattern components identified as contributing to the representational space were subsequently fit using linear regression modeling to the observed similarity in the original ROI (see Fig. 1*D*) to determine the weight of each contributing POI to the observed similarity from the full ROI.

To ensure that regression fits were not a product of overfitting, the following cross-validation procedures were performed: Initial model fitting was performed on a randomly selected sample of participants (“random-sample”: RS = 60), with the identified components fit as a predictor to data from the remaining participants held-out of this initial sample (the “hold-out”: HO = 7). Monte Carlo cross-validation (CV; Picard and Cook, 1984) parameters were chosen to maximize cross-validation performance by minimizing cross-validation variance while maximizing model selection accuracy (Arlot and Celisse, 2010). These analyses identified POIs contributing to representational patterns for each ROI in the RS. Beta coefficients and intercepts, determined by fitting these POIs as predictors to the experimental data, were used to create a reconstructed and averaged dataset. The reconstructed dataset was then fitted as a predictor to the hold-out, with each iteration approximating a single fold of a 10-fold validation.

## Results

**Figure 2.**
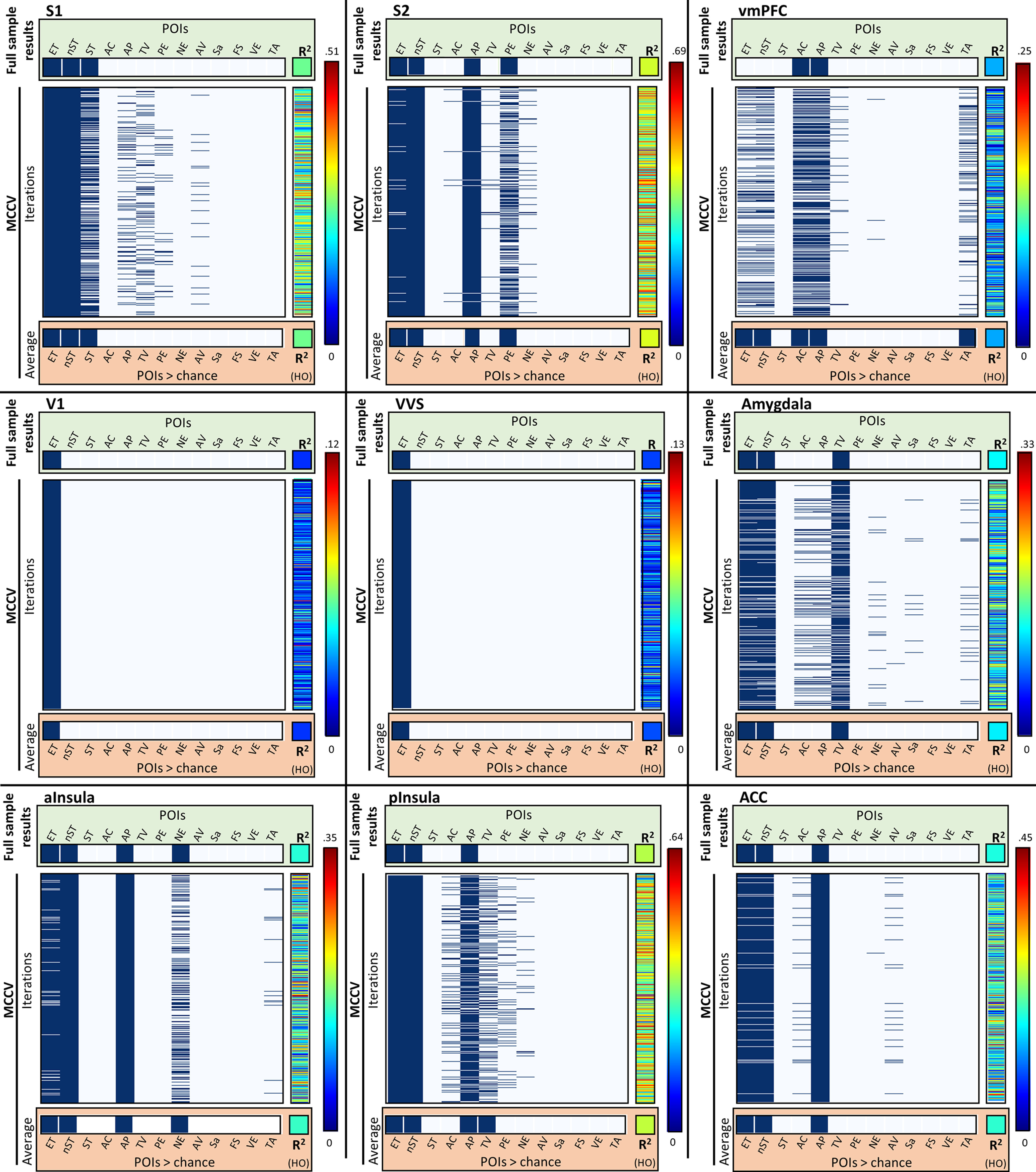
Cross-validation across brains. A 1000 iteration Monte Carlo cross-validation determined (1) that identified POIs from the whole sample data (*n* = 67) were reliably identified when the procedure was replicated on subsets of the sample (*n* = 60) and (2) that reconstructed data generated through POI identification and weighting accurately predicted activational similarity pattern in the held-out participants (*n* = 7). Results from each MCCV iteration are represented as a row of data, with the identified POI noted and the dark blue, and the fit to the HO shown in the center-right column for each ROI. Data summaries collapsed across all MCCV iterations are shown in the red box for each ROI.

**Figure 3.**
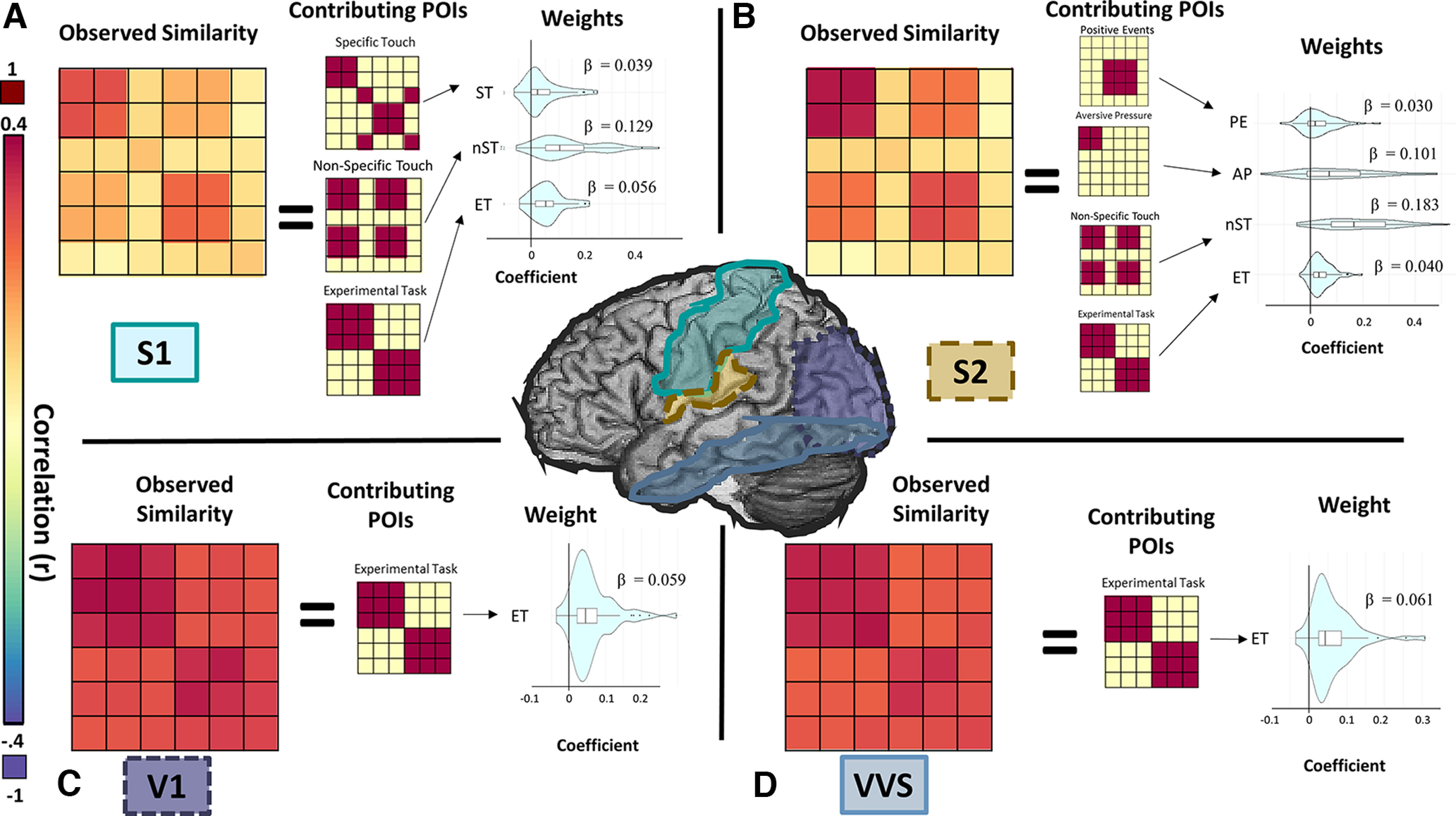
Pattern representation in sensory cortices. For illustrative purposes, this figure presents data from group sample analyses. ***A***, Representational similarity in primary somatosensory cortex (S1) was characterized by POIs indicating representations of discriminatory touch, including nonspecific tactile salience (nST), and specific tactile experience (ST). ***B***, Representation of both hedonic and discriminative tactile signals were observed in S2, with strongest representation of nST and aversive pressure (AP). ***C***, ***D***, Similarity of representations in primary visual cortex (V1) and ventral visual structures (VVS) was characterized by intratask similarity, consistent with the conservation of visual stimuli within experimental tasks.

**Figure 4.**
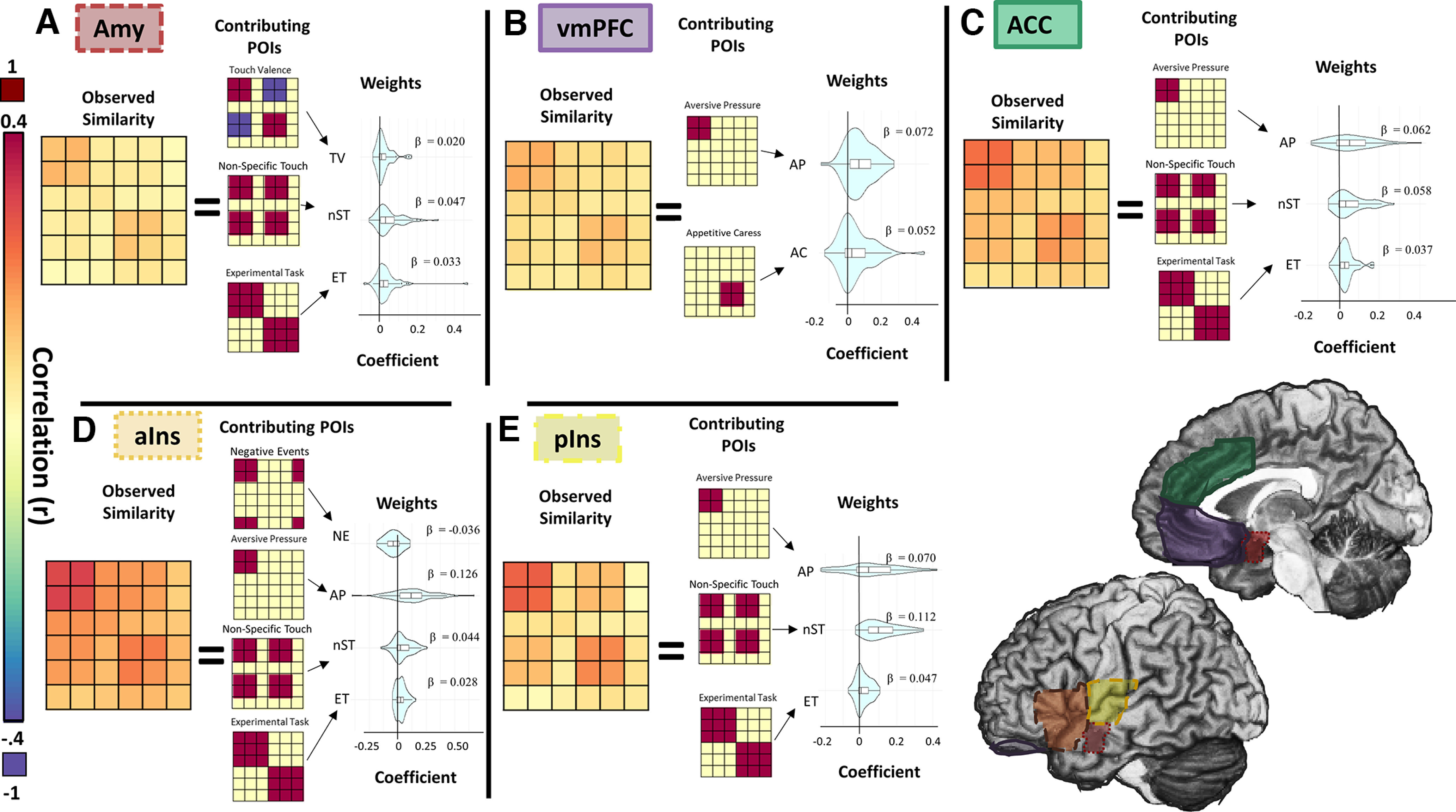
Pattern representations in frontotemporal cortices. For illustrative purposes, this figure presents data from group sample analyses. ***A***, Amygdalae represented POIs consistent with representations of a unidimensional hedonic-tactile spectrum (TV) as well as tactile saliency and general task effects. ***B***, Activation patterns in vmPFC were unique, with representation of appetitive and aversive tactile experience as dissociable contributing components. ***C–E***, Anterior and posterior insula and ACC all displayed patterns of activation consistent with representation of tactile salience and aversive touch, although the specific biases for these types of information varied between the structures.

Results described here are generated from a Monte Carlo cross-validation procedure (1000 iterations) conducted on a random sample of 60 participants (RS = 60). A weighted re-combination of these POIs was then validated against the remaining seven participants (HO = 7; see Fig. 2; Table 3). For detailed presentation of model search paths and component weighting from full sample analyses (i.e., HO = 0; see Figs. 3, 4; Tables 4-Tables 6).

**Table 3:** Cross-validation, average values

ROI	Average n-path	Information pattern component identification – % of simulations (mean contributing β^*a*^; *n* = 55)													HO Fit^*b*^ (*n* = 6, df = 1145)		
		ET	nST	ST	AC	AP	TV	PE	NE	AV	S	FS	VE	TA	*R* ^2^	*p* value	Recon. β
S1	1.934	100 (0.055)	100 (0.129)	60.2 (0.025)	0 -	19.9 -	18.8 -	7.8 -	0 -	4.5 -	0 -	0 -	0 -	0 -	0.248	4.1e-5	0.978
S2	1.607	96.8 (0.041)	100 (0.185)	0 -	1.2 -	98.8 (0.094)	3.3 -	54.8 (0.002)	5.8 -	0 -	0 -	0 -	0 -	0 -	0.411	1.5e-7	1.005
V1	1.212	100 (0.059)	0 -	0 -	0 -	0 -	0 -	0 -	0 -	0 -	0 -	0 -	0 -	0 -	0.021	0.10	1.018
VVS	1.183	100 (0.061)	0 -	0 -	0 -	0.1 -	0 -	0 -	0 -	0 -	0 -	0 -	0 -	0 -	0.028	0.07	1.022
Amy	1.539	85.0 (0.011)	83.4 (0.019)	0 -	20.9 -	22.1 -	75.2 (0.014)	0 -	0.5 -	0.3 -	1.3 -	0 -	0 -	4.7 -	0.116	0.0049	0.988
vmPFC	1.327	28.6 (0.011)	34.9 (0.015)	0 -	65.2 (0.033)	65.7 (0.050)	6.3 -	0 -	0.8 -	0 -	0 -	0 -	0 -	23.1 (0.005)	0.074	0.023	0.934
ACC	1.547	94.4 (0.034)	94.4 (0.055)	0 -	5.6 -	100 (0.067)	0 -	0 -	0.1 -	5.5 -	0 -	0 -	0 -	0 -	0.191	0.00033	0.991
aIns	1.935	91.0 (0.029)	99.8 (0.050)	0 -	0.2 -	100 (0.107)	0 -	0 -	42.2 (−0.016)	0 -	0 -	0 -	0 -	4.5 -	0.153	0.0023	0.969
pIns	2.043	100 (0.043)	100 (0.102)	0 -	16.9 -	83.1 (0.059)	32.6 (0.009)	11.7 -	2.6 -	0 -	0 -	0 -	0 -	0 -	0.363	4.5e-6	0.991

*^a^*Average βs are presented only for POIs identified at a level significantly greater than chance [i.e., proportion of simulations POI is identified > (average # of contributing POIs/Total POIs)].

***^b^***HO fit indicate the average fit across all 1000 Monte Carlo iterations.

**Table 4:** GBFS*-*BIC analysis paths

	Pattern of interest – BIC score															
ROI	None	All	Included+	ET	nST	ST	AC	AP	TV	PE	NE	AV	Sa	FS	VE	TA
S1	−1034.08	−1389.76	-	−1119.00	−1319.74	−1195.35	−1120.78	−1188.25	−1142.20	−1036.30	−1038.53	−1062.32	−1231.40	−1128.35	−952.898	−1027.03
			nST+	−1405.82	-	−1386.70	−1322.49	−1356.94	−1398.61	−1320.73	−1323.06	−1344.03	−1319.99	−1359.95	−1198.08	−1327.22
			nST+ET+	-	-	−1413.71	−1398.70	−1410.78	−1412.63	−1401.79	−1403.37	−1412.35	−1401.70	−1402.46	−1269.41	−1402.46
			nST+ET+ST+	-	-	-	−1408.15	−1413.60	−1410.12	−1406	−1407.64	−1409.97	−1410.12	−1406.57	−1275.21	−1406.57
S2	−779.51	−1455.63	-	−838.18	−1375.29	−938.8	−867.218	−1054.75	−876.23	−776.04	−784.64	−797.25	−1100.80	−862.85	−781.56	−772.60
			nST+	−1441.22	-	−1415.80	−1368.06	−1450.19	−1441.93	−1370.53	−1380.51	−1389.95	−1369.10	−1394.69	−1253.89	−1386.84
			nST+AP+	−1476.14	-	−1457.40	−1463.51	-	−1463.51	−1464.24	−1458.57	−1445.33	−1444.07	−1451.23	−1312.29	−1453.43
			nST+AP+ET+	-	-	−1470.08	−1470.43	-	−1470.43	−1479.58	−1477.12	−1469.88	−1469.05	−1468.89	−1336.99	−1468.89
			nST+AP +ET+PE	-	-	−1472.44	−1473.87	-	−1473.87	-	−1476.96	−1476.96	−1472.41	−1473.82	−1340.98	−1473.82
V1	−579.77	−564.89	-	−604.03	−576.78	−590.49	−576.50	−588.58	−594.09	−573.30	−573.92	−576.08	−576.98	−582.91	−502.20	−580.95
			ET+	-	−599.94	−598.65	−596.78	−601.57	−598.04	−596.83	−597.05	−597.23	−599.05	−596.82	−524.39	−596.82
VVS	−736.73	−735.22	-	−767.83	−730.03	−754.29	−731.47	−746.46	−755.80	−730.06	−732.99	−735.27	−732.87	−744.20	−641.64	−737.98
			ET+	-	−760.75	−764.12	−761.27	−764.98	−762.15	−760.58	−761.90	−761.75	−761.95	−760.89	−669.51	−760.89
Amy	−2239.68	−2372.46	-	−2322.09	−2317.26	−2314.00	−2276.71	−2323.08	−2330.33	−2238.72	−2238.99	−2253.95	−2280.78	−2273.06	−2028.50	−2247.58
			TV+	−2337.51	−2388.73	−2330.90	−2325.83	−2348.95	-	−2324.69	−2324.55	−2331.39	−2360.47	−2325.17	−2112.81	−2326.49
			TV+nST+	−2400.95	-	−2381.51	−2385.18	−2385.18	-	−2382.65	−2382.53	−2387.46	−2381.51	−2381.50	−2150.11	−2395.95
			TV+AT+ET+	-	-	−2393.94	−2397.44	−2397.44	-	−2393.74	−2393.72	−2393.90	−2393.94	−2396.33	−2161.91	−2396.33
vmPFC	−2292.75	−2366.00	-	−2340.14	−2348.15	−2327.19	−2309.24	−2354.44	−2345.23	−2286.66	−2293.21	−2297.90	−2312.54	−2305.47	−2108.47	−2298.28
			AP+	−2370.85	−2371.35	−2355.95	−2389.60	-	−2362.61	−2358.72	−2358.69	−2347.65	−2354.81	−2350.36	−2149.25	−2358.10
			AP+AB+	−2387.28	−2384.78	−2384.56	-	-	−2384.78	−2383.20	−2387.06	−2388.71	−2382.39	−2383.69	−2177.70	−2391.48
ACC	−2050.26	−2292.87	-	−2123.23	−2213.52	−2132.29	−2071.41	−2224.68	−2137.06	−2043.06	−2060.56	−2059.25	−2131.92	−2086.43	−1893.75	−2052.87
			AP+	−2240.72	−2289.11	−2231.99	−2284.46	-	−2227.37	−2233.28	−2253.11	−2217.89	−2250.82	−2222.83	−2036.43	−2224.99
			AP+nST+	−2317.67	-	−2288.41	−2299.75	-	−2299.75	−2289.31	−2292.42	−2281.94	−2281.96	−2283.94	−2087.96	−2305.39
			AP+nST+ET+	-	-	−2310.66	−2310.88	-	−2310.88	−2311.97	−2314.83	−2310.52	−2311.50	−2313.75	−2115.92	−2313.75
aIns	−1387.27	−1569.49	-	−1435.42	−1496.30	−1434.02	−1387.97	−1540.58	−1441.83	−1380.11	−1394.42	−1390.16	−1435.53	−1408.01	−1261.73	−1387.94
			AP+	−1545.03	−1572.27	−1536.46	−1560.92	-	−1535.32	−1543.32	−1567.10	−1535.10	−1548.12	−1534.66	−1374.58	−1539.18
			AP+nST+	−1582.81	-	−1565.64	−1569.63	-	−1569.63	−1569.94	−1580.08	−1565.43	−1565.74	−1565.25	−1393.49	−1580.58
			AP+nST+ET+	-	-	−1577.88	−1575.91	-	−1575.91	−1576.93	−1585.17	−1576.70	−1577.40	−1579.21	−1404.64	−1579.21
			AP+nST +ET+	-	-	−1578.81	−1577.93	-	−1577.93	−1578.11	-	−1578.11	−1578.97	−1578.93	−1405.69	−1578.93
pIns	−1741.87	−2319.59	-	−1841.54	−2183.59	−1919.5	−1821.63	−2034.84	−1884.29	−1737.7	−1755.68	−1768.59	−1983.73	−1830.78	−1672.32	−1736.80
			nST	−2294.65	-	−2247.15	−2177.40	−2289.92	−2296.49	−2178.27	−2198.34	−2207.35	−2176.96	−2212.04	−2018.71	−2212.67
			nST+ET	-	-	−2295.48	−2297.78	−2342.33	−2311.86	−2287.41	−2300.14	−2299.12	−2287.55	−2287.41	−2119.58	−2287.41
			nST+ET+AP	-	-	−2306.10	−2323.47	-	−2323.47	−2307.27	−2297.62	−2286.37	−2283.35	−2293.74	−2106.30	−2305.95

Provided are the most likely combination of pattern component models (POIs) to explain observed representational patterns for each ROI. Model fitting was performed by identifying the best individually fitting POI, then iteratively adding remaining POIs. POIs held from prior levels of analyses are indicated in under “Included+” (forth column from left), with the BIC score resulting from combining the held POIs independently with all remaining POIs displayed in the right columns. Significantly improved fit because of the addition of an additional POI is indicated by ΔBIC > 2. Note, identified final POI combinations predicted the observed data significantly better than a combination of all POIs (“All”), and a single uniform predictor (“None”).

**Table 5:** Alternate GBFS-BIC paths: S1 and pIns

	Pattern of interest – BIC score															
ROI	None	All	Included+	ET	nST	ST	AC	AP	TV	PE	NE	AV	Sa	FS	VE	TA
S1	−1034.08	−1389.76														
			-	−1119.00	−1319.74	−1195.3	−1120.78	−1188.25	−1142.20	−1036.30	−1038.533	−1062.32	−1231.40	−1128.35	−952.89	−1027.03
			AP+	−1405.82	a	−1386.70	−1322.49	−1356.94	−1398.61	−1320.73	−1323.06	−1344.03	−1319.99	−1359.95	−1198.08	−1327.22
			AP+nST+	b	a	−1413.71	−1398.70	−1410.78	−1412.63	−1401.79	−1403.37	−1412.35	−1401.70	−1402.46	−1269.41	−1402.46
			AP+nST+TV+	b	a	−1410.12	−1409.90	−1409.90	c	−1405.47	−1405.93	−1407.24	−1410.12	−1406.74	−1275.21	−1406.74
pIns	−1741.87	−2319.59	-	−1841.54	−2183.59	−1919.50	−1821.63	−2034.84	−1884.29	−1737.77	−1755.68	−1768.59	−1983.73	−1830.78	−1672.32	−1736.80
			nST+	−2294.65	a	−2247.15	−2177.40	−2289.92	−2296.49	−2178.27	−2198.34	−2207.35	−2176.96	−2212.04	−2018.71	−2212.67
			nST+TV+	−2311.86	a	−2289.91	−2323.47	−2323.47	b	−2298.02	−2289.78	−2295.92	−2289.91	−2289.50	−2118.30	−2301.72
			nST+TV+AB+	−2339.41	a	−2316.92	c	−2323.47	b	−2317.21	−2331.62	−2323.07	−2316.92	−2316.49	−2137.58	−2329.016
			nST+TV+AB+ET+	d	a	−2332.17	c	−2339.41	b	−2337.65	−2339.91	−2332.34	−2332.17	−2333.32	−2152.38	−2333.32
			-	−1841.54	−2183.59	−1919.50	−1821.63	−2034.84	−1884.29	−1737.77	−1755.68	−1768.59	−1983.73	−1830.78	−1672.32	−1736.80
			nST+	−2294.65	a	−2247.15	−2177.40	−2289.92	−2296.49	−2178.27	−2198.34	−2207.35	−2176.96	−2212.04	−2018.71	−2212.67
			nST+TV+	−2311.86	a	−2289.91	−2323.47	−2323.47	b	−2298.02	−2289.78	−2295.92	−2289.91	−2289.50	−2118.30	−2301.72
			nST+TV+AP+	−2339.41	a	−2316.92	−2323.47	c	b	−2317.21	−2331.62	−2323.07	−2316.92	−2316.49	−2137.58	−2329.01
			nST+TV+AP+ET+	d	a	−2304.62	−2339.41	c	b	−2308.88	−2307.63	−2304.79	−2304.62	−2305.74	−2132.64	−2305.74

With no clear POI providing optimum fit at the 2nd and 3rd level of GBFS-BIC analyses (i.e., POI_1_ – POI_2_ < 2), search paths for each statistically equivalent option were completed. The POI combination providing the lowest BIC at completion of the path was defined as the best fitting combination.

**Table 6:** POI coefficient weights: full sample

	Regression		Information pattern component weighting – β coefficients														
ROI	*R* ^2^	*p* value	ET	nST	ST	AC	AP	TV	PE	NE	AV	S	FS	VE	TA	lST	rST
S1	0.247	2.8e-50	0.056	0.128	0.039	-	-	-	-	-	-	-	-	-	-	n/a	n/a
lS1	0.237	0.00053	0.051	0.136	-	-	0.045	-	-	-	-	-	-	-	-	n/a	-
rS1	0.240	7.3e-7	0.067	0.131	-	-	-	-	-	-	-	-	-	-	-	0.050	n/a
S2	0.403	9.1e-88	0.040	0.183	-	-	0.101	-	−0.030	-	-	-	-	-	-	n/a	n/a
V1	0.021	2.0e-8	0.059	-	-	-	-	-	-	-	-	-	-	-	-	n/a	n/a
VVS	0.026	6.1e-10	0.061	-	-	-	-	-	-	-	-	-	-	-	-	n/a	n/a
Amy	0.120	0.00036	0.033	0.046	-	-	-	0.020	-	-	-	-	-	-	-	n/a	n/a
vmPFC	0.075	8.9e-21	-	-	-	0.052	0.075	-	-	-	-	-	-	-	-	n/a	n/a
ACC	0.184	1.1e-10	0.037	0.058	-	-	0.062	-	-	-	-	-	-	-	-	n/a	n/a
aIns	0.147	8.2e-13	0.028	0.044	-	-	0.126	-	-	−0.036	-	-	-	-	-	n/a	n/a
pIns	0.356	6.0e-66	0.047	0.112	-	-	0.070	-	-	-	-	-	-	-	-	n/a	n/a

### POI identification and weighting

To determine the combinations of POIs that best explained the observed patterns of data for each ROI, PCM was conducted through Bayesian information criterion (BIC) analyses and an uninformed greedy best-first search (GBFS) algorithm. This strategy of iterative model fitting with progressively increasingly complex predictor combinations contrasts previous RSA approaches which typically do not combine potential predictors (Kragel et al., 2018; Dobs et al., 2019). Through the current analyses, we are able to determine the degree information was integrated versus separate in any specific region. To ensure that regression fits were not a product of overfitting, we performed Monte Carlo cross-validation. Specific outputs of interest included the proportion of cross-validation iterations (i = 1000) in which a POI was identified as a contributing component of the experimental data in the random samples, the representational weight of those components identified at a rate significantly greater than chance, and the model fit of the reconstructed RS components to the holdout (Fig. 2; for complete summary of the cross-validation results, see Table 3). The proportion of iterations for POI identification was compared with chance identification for each region of interest (ROIs; i.e., number of POIs identified/total number of POIs). For each iteration of the MCCV procedure, the total number of paths required for a given search is defined as the n-path.

#### Sensory regions of interest

##### Primary somatosensory cortex (S1)

In S1, a POI modeling nonspecific aspects of tactile experience (nST) was the strongest individual predictor of S1 representational patterns in the random samples (β_nST_ = 0.129). Additional POIs identified contributing to overall representational patterns in S1 modeled experimental task and nonhedonic tactile experience (β_ET_ = 0.055 and β_ST_ = 0.025, respectively). In the held-out sample, POIs identified in the random sample explained 24.8% of the variance (*R*^2^ = 0.248, *F*_(1,145)_ = 52.13, *p *<* *0.001). This pattern indicates a representation of distinct discriminative tactile experiences rather than hedonic value in S1 (Fig. 3*A*).

Two additional PCM analyses were conducted in S1 ROIs that interrogated right and left S1 ROIs independently. Importantly, these analyses added two additional POIs, modeling the left and right lateralized components of nonhedonic tactile experience. In a pattern similar to that observed across the bilateral ROI, data from the random sample were predicted most strongly by POIs modeling nonspecific aspects of tactile experience (Left S1: β_nST_ = 0.140; Right S1: β_nST_ = 0.129), with secondary contributions from the POI modeling global similarity within, but not between, experimental tasks (Left S1: β_ET_ = 0.052; Right S1: β_ET_ = 0.066). This similarity, however, was not observed for representations of nonspecific tactile experience observed in the bilateral ROI. Left S1 did not represent aversive pressure as isolated from other forms of nonhedonic tactile states (β_AP_ = 0.032) and was lacking general representation for right lateralized nonhedonic touch. By contrast, right S1 represented nonhedonic touch experience (i.e., appetitive caress and scanner-generic touch as distinct states β_lST_ = 0.045). In both unilateral ROIs, POIs contributing to representational patterns in the random sample also significantly predicted the representational patterns of the held-out participants (Left S1: *R*^2^ = 0.237, *F*_(1,145)_ = 49.68, *p *<* *0.001; Right S1: *R*^2^ = 0.240, *F*_(1,145)_ = 49.97, *p *<* *0.001).

##### Secondary somatosensory cortex (S2)

For S2, the strongest predictor of representational patterns was nonspecific touch (β_nST_ = 0.185). Additional POIs identified as contributing to overall representational patterns were aversive touch (β_AP_ = 0.094), experimental task (β_ET_ = 0.041), and the task-specific positive experience (i.e., caress, or safety; β_PE_ = 0.002). Combined, weighted POIs identified as being represented in the random sample accounted for an average of 41.1% of the variance in the held-out participants (*R*^2^ = 0.411, *F*_(1,145)_ = 113.07, *p *< 0.001). This suggests that S2 may receive hedonic signals that are not represented in S1 (Fig. 3*B*).

##### Visual cortices

The most predictive POI in both V1 and ventral visual stream modeled task-related changes in experience. No other POIs were identified at a rate significantly greater than chance. In V1, this component explained an average of 5.9% of the variance in the initial random sample, while in VVS, it explained an average of 6.1% of the variance. This weighted component, however, failed to significantly predict patterns observed in the held-out participants in cross-validation procedures (V1: *R*^2^ = 0.021, *F*_(1,145)_ = 4.27, *p *=* *0.10; VVS: *R*^2^ = 0.028, *F*_(1,145)_ = 5.27, *p *=* *0.07). This suggests that representational patterns in visual cortices may reflect visual attentional demands of the experimental task and are relatively uninformative of nonvisual information, regardless of its hedonic value (Fig. 3*C*,*D*). The inability to replicate significant findings in the held-out participants, however, suggests that activational patterns in these regions are likely not driven by information modeled in the current set of POIs, but may instead be more accurately represented by untested representational patterns related to specific aspects of visual (rather than tactile) experience

#### Integrative regions of interest

##### Amygdalae

A combination of three POIs were identified as contributing to the representational patterns of bilateral amygdalae in the initial random sample. These POIs modeled a linear spectrum of tactile valence (β_TV_ = 0.014), nonspecific tactile experiences (β_nST_ = 0.019), and global differences in experimental tasks (β_ET_ = 0.011), respectively. POIs identified in the random sample accounted for 11.6% of the observed variance in the held-out participants (*R*^2^ = 0.116, *F*_(1,145)_ = 20.86, *p *=* *0.0049). Notably, activation patterns in the amygdalae represented valence on a linear spectrum, where appetitive and aversive touch were most dissimilar, polar opposites of a shared representational space (Fig. 4*A*).

##### Ventromedial prefrontal cortex

In the vmPFC, representational patterns were predicted by multiple POIs, most notably the two distinct hedonic touch POIs: Aversive pressure (β_AP_ = 0.050) and Appetitive caress (β_AB_ = 0.033). Additional POIs identified as contributing to the representational patterns in the random sample modeled differences in experimental task (β_ET_ = 0.011), nonspecific tactile representations (β_nST_ = 0.015), and temporal order of trial events (i.e., whether or not the similarity matrix reflected temporally adjacent events; β_TA_ = 0.005). Total variance accounted for in the held-out participant by the models identified in the random samples was on average 7.4% (*R*^2^ = 0.074, *F*_(1,145)_ = 12.98, *p *=* *0.023). This demonstrates that vmPFC activity contains information about the hedonic value of the tactile stimulation, representing positive and negative values as distinctly independent and nonopposing signals (Fig. 4*B*). Furthermore, the heterogeneity of nonhedonic POI identification in this region this suggests that while vmPFC does consistently represents aversive pressure and appetitive caress, there may be extensive interparticipant variability regarding what information can be in this region.

##### Anterior cingulate cortex

The aversive pressure (AP) POI was the strongest predictor of ACC activation patterns (β_AP_ = 0.067). Additional POIs identified as contributing to the representational patterns included those modeling nonspecific tactile experience (β_nST_ = 0.055) and experimental task (β_ET_ = 0.034). A weighted combination of the POIs identified in the random samples predicted an average of 19.1% of the variance in the held-out participants (*R*^2^ = 0.191, *F*_(1,145)_ = 37.84, *p *<* *0.001). This suggests that the ACC represents general tactile information but is particularly sensitive to tactile information associated with pain (Fig. 4*C*).

##### Insula

The insula was anatomically subdivided at the anterior commissure into distinct nonoverlapping anterior/posterior regions. Representational patterns in the anterior insula (aIns) were significantly predicted by four POIs. In order of representational strength, these POIs modeled aversive tactile experience (β_AP_ = 0.107), nonspecific tactile experience (β_nST_ = 0.050), experimental task (β_ET_ = 0.029), and task-contextualized negative events (i.e., aversive touch OR lack of appetitive touch; β_NE_ = −0.016). POIs identified in the random sample predicted an average of 15.3% of the variance in the held-out participants (*R*^2^ = 0.153, *F*_(1,145)_ = 28.74, *p *<* *0.0023).

As in the aIns, in pIns, activity was significantly predicted by POIs modeling aversive tactile experience (β_AP_ = 0.059), nonspecific tactile experience (β_nST_ = 0.102), and experimental task (β_ET_ = 0.043). An additional POI modeling tactile valence (β_TV_ = 0.009) was also identified in this region. In pIns, combinations of POIs identified in the random sample predicted an average of 36.3% of the variance in the held-out participants (*R*^2^ = 0.363, *F*_(1,145)_ = 90.82, *p *<* *0.001). This demonstrates that whereas the general type of information processed across the insula may be similar for the anterior and posterior sections, each region sensitive to both hedonic and nonhedonic signals, the precise nature and dominance of these representations differs (Fig. 4*D*,*E*).

### n-Path analyses

To assess the robustness of POI contributions, a one-way ANOVA was conducted on the average number of search paths required to find the best fitting component combination for each iteration (i.e., n-path data). This identified a significant main effect of region (F_(8,7992)_ = 195.507, *p < *0.001). A follow-up series of independent sample *t* tests (all reported *p* values are Bonferroni-corrected) identified four distinct clusters of ROIs characterized by their n-path. A lower search path likely indicates either a poor fit of the POI models (if only a single model is frequently identified; e.g., V1/VVS), or robust representations for a specific subset of models (if identified POI > 1; e.g., vmPFC). By contrast, a higher n-path likely indicates more overlapping representational space (e.g., insular subdivisions). Visual areas required expansion of fewer paths than any other area (all *p *<* *0.001), yet they did not differ significantly from each other (*p *=* *1.0). vmPFC had more branching than either V1 or VVS but less than all other ROIs (all *p *<* *0.001). ACC, amygdala, and S2 did not significantly differ from each other, yet required less expansion of search paths than S1 or either insular ROI (all *p*s* *<* *0.001). Finally, while the anterior insula did not significantly differ from either the posterior insula or S1 (both *p*s* *=* *1), the posterior insula displayed greater branching than S1 (*p *<* *0.001). The greater n-paths in these regions suggests that there is likely greater overlap of representational space in these regions between the modeled components compared with representations in other regions.

## Discussion

In this study, we applied a novel form of pattern component modeling with representational similarity analysis to demonstrate that by fitting predefined representational patterns (POIs) to observed patterns of similarity between voxel activation patterns in the brain, we are able to model the specific nature of information represented in a brain region, teasing apart isolated or integrated signals of valence and experience. Specifically, in the current work, we were able to dissociate how discriminatory versus hedonic tactile information, coded at the somatosensory receptors and carried by A- and C-/CT-fibers, respectively, contribute to population coded representations in the human brain. Distinct representations of hedonic information were observed in frontal and temporal structures, including ventromedial prefrontal cortex (vmPFC), insula (Ins) and anterior cingulate cortex (ACC), as well as in secondary somatosensory cortex (S2). Primary somatosensory cortex (S1) did not represent all tactile information coded by peripheral receptors, with limited representation of some hedonic signaling. Specifically, we did not observe any representation of positive hedonic touch signals and only limited representation of negative hedonic signals (carried by CT-fiber afferents and C-fiber afferents, respectively. Visual areas, including primary/secondary visual cortex (V1) and ventral visual structures (VVS), displayed no representation of either affective or discriminative touch information.

Together, the findings support the hypothesis that processing of sensation carried by hedonic-labeled tactile signals from C- and CT-fiber pathways, despite their salience and homeostatic significance, does not depend on representation of these signals in S1. Rather, this information is represented predominantly in frontotemporal structures more typically implicated in interoception (Craig, 2011; Pollatos et al., 2016; Strigo and Craig, 2016) and the central mediation of emotional relevance (McFarland and Sibly, 1975; Rolls, 2000; Todd et al., 2020). Of note, however, negative hedonic information made a minor contribution to representational patterns in S1 contralateral to the tactile stimulation, indicating that some nociceptive information does reach this area independent of frontotemporal processing. Thus, some propagation of tactile hedonic information is distinct from traditional exteroceptive signals highlighting nontraditional mechanisms (Kryklywy et al., 2020) by which prioritized information may be incorporated into emotionally-guided cognitive processes.

### Cortical representations for nonhedonic touch

In S1, neural activity displayed representational patterns that discriminated tactile experiences, as well as nonvalence specific components, of tactile manipulations. The finding of strong representation of specific touch experiences in this area are consistent with S1’s traditional role in exteroception, processing discriminatory tactile information as carried by out A-fiber afferents (McGlone and Reilly, 2010; McGlone et al., 2014). The POI “specific touch” is defined such that representational patterns for trials with no hedonic manipulation (i.e., the somatosensory experience generated by lying in a scanner) have equivalent contribution to the global representation of touch experience as trials with hedonic manipulation. Thus, its manifestation is unlikely to be generated by peripheral hedonic signaling alone. In addition to specific touch, S1 strongly represented the experience of “nonspecific touch” indicating activity in this region was also influenced by salient tactile experiences and contained shared representational space for both appetitive and aversive tactile manipulations. This suggests that these representations are likely not shaped by information carried by C- and CT-fiber in isolation, as the two distinct peripheral signals are represented by overlapping activation representational patterns. Nonspecific touch representation is likely to be either a discriminatory representation of body location (i.e., arm; not dependent on C- or CT-fiber activation) or general tactile salience (may or may not integrate information from C- and CT-fiber activation; i.e., hedonic salience). In support of the latter interpretation, there is evidence that S1 likely integrates re-entrant hedonic signals from multisensory emotion-related regions (Vuilleumier, 2005; Pessoa and Adolphs, 2010; Orenius et al., 2017).

Components indexing unprocessed projections of hedonic-labeled afferent pathways (i.e., “aversive pressure” and “appetitive caress”) were absent in bilateral representational patterns observed in S1, which may on first inspection be interpreted as hedonic signals not being instantiated in traditional somatosensory processing structures. Yet, unilateral investigation of left S1 identified representation patterns associated with aversive pressure in absence of specific touch experience, indicating that discriminative and nociceptive information may be integrated before reaching S1 (for candidate regions, see Abraira et al., 2017; Marshall and McGlone, 2020; Neubarth et al., 2020). Alternatively, it may be that sustained changes in tonic firing of rates of slow adapting mechanoreceptors (for review, see Knibestol, 1975; Abraham and Mathew, 2019) in response to the strong pressure manipulation (right hand), result in a distinct tactile representation in left S1 during CS− trials (pressure task). This representation would be distinct from the representation of scanner-generic sensation (right hand) experienced during the caress (which was applied to the left hand). Given substantive evidence to support both interpretations, it is likely that the observed POI contributions reflect a combination of these processes. No evidence of unique representational patterns in S1 for signals of appetitive hedonic information was observed.

Taken together, nonhedonic tactile representation, likely of signals carried along A-fiber pathways (McGlone and Reilly, 2010), appear to dominate activity in early somatosensory cortices. While some evidence for hedonic representations in these regions exists, it appears that precortical integration of A- and C-/CT-fiber pathways (Abraira et al., 2017; Marshall and McGlone, 2020; Neubarth et al., 2020), or re-entrant feedback from higher order integrative structures (Vuilleumier, 2005; Pessoa and Adolphs, 2010) are the most probable source of these representations.

### Cortical representations for hedonic touch

Amongst all regions investigated, only the vmPFC displayed independent representation of both appetitive and aversive touch (POIs: AC/AP). This suggests that this region either (1) receives information carried along C- and C-tactile fiber afferents as distinct signals before their integration with each other or other tactile information, or (2) has decomposed an integrated hedonic representation back into distinct signals of positive and negative value to inform situation specific behaviors and decision. The potential of first order representation of peripherally labeled hedonic signals in vmPFC is particularly intriguing considering the critical role these ventral medial structures play in appraising emotional salience to guide value-based decision-making (Euston et al., 2012; Dixon et al., 2017; Hiser and Koenigs, 2018). Propagation of hedonic-labeled tactile signals to these regions independent of any prior cortical processing would act as a mechanism to facilitate the prioritization of evolutionarily relevant sensation (Kryklywy et al., 2020), and allow for expedited integration of action-outcomes into value appraisal to guide decision-making processes.

The absence of distinct representations for pleasurable tactile signals in both the anterior and posterior insula is notable, as these regions have been highlighted as potential cortical recipients of C-tactile fiber signaling (Olausson et al., 2002; Rolls et al., 2003). While this may be because of variability in response to the appetitive touch manipulation used in the current design, the identification of a clear appetitive caress contribution to representational patterns in vmPFC indicated that this is unlikely. It is important to note much of the prior work identifying modulation of insula activity by pleasurable touch has been performed either independent of aversive touch (Olausson et al., 2002), or examined by treating the two signals orthogonally without direct comparison (Rolls et al., 2003). This leaves open the possibility that previous results were driven by general affective salience of the tactile cue (as observed in the current work), rather than pleasurable sensation alone. Related to this, the potential for sensory adaptation or habituation to hedonic tactile experience should be considered (McBurney and Balaban, 2009; Morrison, 2016). The current design relies on the averaged representational similarity across six distinct tactile exposures for each condition. Thus, it is possible that differences in the time course of downregulating tactile signaling between painful and pleasurable stimulation following multiple exposures may underlie some of the differences in representational strength of hedonic information. This question could be targeted by future studies that dissociate early and late exposure to hedonic touch.

Although distinct representation of appetitive touch was identified only in the vmPFC, distinct representations of aversive pressure were identified within the ACC as well as the anterior and posterior insula. Notably, both of these regions are heavily implicated in the representation of painful experience (Corradi-Dell’Acqua et al., 2016; Kragel et al., 2018) and are postulated to underlie awareness of one’s own internal homeostatic balance (Craig, 2011, 2015; Pollatos et al., 2016; Strigo and Craig, 2016). One potential explanation for this pattern of results is that hedonic-labeled peripheral afferents are not processed as tactile signals in the traditional view of sensation (Pinel and Barnes, 2018; Gazzaniga et al., 2019). That is, they may not be instantiated in neocortex as representing the experience of contact with external objects in the environment. Rather, information carried along these pathways indicates internal concerns about homeostatic threat or social safety (Craig, 2011, 2015) and manifest cognitively as emotional feelings congruent with these states. Information about the internal state acts can then act as an immediate mechanism for motivating response, independent of its representation as an exteroceptive tactile experience or any other form of cognitive processing.

### Integrated representation of tactile experience

Multiple regions displayed patterns of activity that integrated signals from both C- and CT-fiber afferents. These representational patterns showed distinct similarity/dissimilarity between hedonic conditions, rather than maintaining the independence of their sources, indicative of prior processing and representational integration of this information. Specifically, in the ACC as well as the anterior and posterior insula, patterns of neural activity were found to represent nonspecific touch beyond those identified for aversive pressure. These integrated representations likely indicate that processing of sensory information has occurred before their affective representation the insula and ACC, in a manner consistent with traditional models for emotional prioritization (Rolls, 2000, 2019; Vuilleumier, 2005; Pessoa and Adolphs, 2010).

While none of the anterior or posterior insula, ACC, or vmPFC were found to display opposing representations of hedonic valence, in the amygdala, a distinct representation of the hedonic experience of touch valence was observed. Here, signals of tactile valence were represented as a single linear vector, with positive and negative hedonic conditions represented as polar ends of a single valence spectrum. Thus, before its representation, or as part of its processing in the amygdala, tactile information must be integrated into the same representational space. These amygdalar bi-polar valence representations are consistent with those identified in the olfactory domain (Jin et al., 2015), but have not been reported for either gustatory or visual hedonic information (Chikazoe et al., 2014, 2019). This divergence indicates a probable modal-specificity of hedonic processing in the amygdala rather than a centralized a-modal representation of emotional information (Miskovic and Anderson, 2018). In the current study, this unidimensional hedonic vector may be related to the association of the tactile sensation with the concurrent visual stimuli rather than the tactile signals in isolation, as multiple studies in both humans and nonhuman primates have implicated this region in guiding affect-biased attention (Todd et al., 2020) and emotional learning in vision (Morris et al., 1998; Everitt et al., 2003). Of note, however, there is substantial evidence to suggest that both hedonic responding and attentional biases may be heavily influenced by individual differences between subjects (Nielsen et al., 2009; Todd et al., 2012; Harjunen et al., 2017). Future work conducted in a larger sample population could provide additional insight into this possibility.

### Analytic considerations

By iteratively fitting combinations of predefined models of representational patterns to observed data, rather than fitting each pattern in isolation, we were able to interrogate the representation of information with more conceptual resolution than previous work. Specifically, this approach allows researchers to determine whether individual brain regions represented information as either isolated or integrated patterns, as well as the strength of each representational pattern in the context of others. Applying this technique to targeted hypothesis-specific representations, we were able to identify meaningful differences in the cortical representation of discriminative versus hedonic somatosensory signals.

It is notable that with the current implementation, all patterns of interest were defined as matrices of either 0 or 1 (i.e., perfect or no correlation). While the reliability of results we obtained (see the cross-validation procedures), and their consistency to hypothesized functioning in the tested regions (Craig, 2011, 2015; Cauda et al., 2012; Jin et al., 2015; Dixon et al., 2017; Miskovic and Anderson, 2018), support its implementation in this form, future work using similar technique may look to add additional refinement to these models. In many cases, there may be a well-supported reason to expect partial, or scaled correlation between conditions, rather than absolutes. While the coarse approach to modeling implemented in the current work does provide meaningful insight into the nature of tactile representation, further refinement of the representational pattern investigated may provide additional details to this picture.

An additional limitation to the current work that by constraining our search to a greedy best-first approach, we have by algorithmic definition limited the combinations of POIs tested. We believe that our current approach provides greater refinement toward understanding how multiple sources of information contribute in parallel to observed neural representational patterns than previous approaches, and does so in a computationally efficient manner. That said, we also acknowledge the importance of future work to explore and compare alternate search algorithms as well as computationally intense brute-force approaches. We believe that POI-guided pattern component modeling represents an exciting new approach to multivariate analyses, and that its application to human neuroimaging data can allow for increasingly detailed “read out” into how and where information in processed within the human brain.

In conclusion, in this work, we have outlined an exciting extension of traditional representational similarity analyses. Using this approach, we demonstrated that hedonic tactile information is not processed in the same fashion as nonhedonic tactile information. The full spectrum of hedonic tactile information, signals carried in the periphery by C- and CT-fiber pathways, is not represented in primary sensory cortices but is represented in some frontotemporal structures typically associate affective processing. Additionally, we believe that the POI-based approach to pattern component modeling outlined here reflects an exciting new avenue for future multivariate neuroimaging analyses. It provides a tool to decompose observed patterns of representational similarity into intuitive, theory-guided representational subcomponents (i.e., POIs), thus allowing researcher to probe deeper into the content of information instantiated by the brain.

## Synthesis

Reviewing Editor: Alexander Soutschek, Ludwig-Maximilians-Universitat Munchen Decisions are customarily a result of the Reviewing Editor and the peer reviewers coming together and discussing their recommendations until a consensus is reached. When revisions are invited, a fact-based synthesis statement explaining their decision and outlining what is needed to prepare a revision will be listed below. The following reviewer(s) agreed to reveal their identity: Maximilian Riesenhuber.

Reviewer 1

Enthusiasm is high for the topic of the study, that of processing of somatosensory (SS) stimulation, with an fMRI investigation of differential responses across the brain to hedonic, aversive and stimuli without a particular valence. This is an understudied field, and much of the cortical processing of hedonic vs. aversive SS stimuli is still poorly understood.

The enthusiasm for the topic is somewhat balanced (but not outweighed) by concerns about the data analyses. In particular, the approach used in the paper is a bit too sophisticated, consisting of RSA with mRDMs (it is not quite clear to me why the manuscript uses non-standard terms such as POI for mRDM -- this makes the manuscript more opaque than it needs to be for someone with knowledge of RSA), with a technique that aims to represent nRDMs through a weighted combination of mRDMs reflecting different neural tuning models (e.g., an mRDM reflecting neural representations coding for valence, for specific touch etc. etc.). There are a couple of concerns with the technique: One is the sheer number of mRDMs -- there are 13 different mRDMs for different possibilities of neuronal tuning. Given that the mRDMs are not orthogonal, this creates the problem that there are multiple possible fits for a given nRDM. The authors introduce a greedy fitting strategy, which is sensible in terms of being computationally feasible, but the problem persists that there might be other fits than the ones shown that are either better or at least equally good but using different mRDM components. This problem of arbitrariness/non-uniqueness of fits is exacerbated by the arbitrariness associated with the mRDMs themselves: For instance, in the “negative events” mRDM, aversive stimulation is weighed as much as the absence of an appetitive stimulation when expected. These are debatable, ad hoc choices, and it is unclear how different component weights here and in other matrices would change the results. These concerns about the rigor of the analyses reduces the study’s potential impact, and it would correspondingly strengthen the study if the authors could show that their findings are robust with respect to different choices in mRDM or fitting procedures. At the least, the authors should address the arbitrariness of the mRDMs/POIs and the non-uniqueness of solutions and how this affects the strength of their conclusions in the Discussion. There are some other concerns, in particular regarding the high rate of subjects whose data could not be processed: Out of 121 participants, preprocessing was not completed for 54 of them, including 12 for which data files were missing. These are somewhat high percentages and it would be good to better understand the reason for the large number of subjects for which preprocessing failed. Yet, the final dataset of 67 participants still seems sufficiently large.

In summary, even with these concerns, given the understudied nature of SS processing mechanisms in the human brain and the study’s combination of valent and non-valent stimuli, with the changes suggested above, this study is an interesting addition to the literature!

Minor:

l. 22: should be “emphasize”

l. 59 should be “differentiate”

l. 202: Lombardo et al. 2016 reference missing from reference list

l. 269: reference formatting messed up

l. 471: should be “represent”

Reviewer 2

The authors use a subset of a large-scale fMRI dataset to present a variation of pattern-component modeling for a-priori hypothesis testing which involves searching for “patterns of interest”, or POIs, in various brain regions. The authors use this method to investigate representation of tactile information in the brain. Overall the subject of the manuscript is interesting and the manuscript is well written. My main concern for the manuscript is that it is difficult to figure out whether it is a methods paper or a research paper.

Main:

1. I am still confused about exactly how new the implementation of PCM used in this manuscript is, and how important the authors believe this aspect of the paper to be. On one hand, the authors state that they developed and applied it here, but there are also some citations that make it seem like it already exists. In general, if the focus of the paper were on the method, I would expect more validation and more introduction of the method in the manuscript. Instead, the method is introduced in the methods section only -- and even there, the focus is more on the composition of the POIs than the implementation of the method. However, in the Conclusion and Abstract, the main contribution seems to be the development or extension of the method rather than the conclusions of the fMRI study analyzed. It would be helpful if the authors clarified their intentions by making the emphasis more consistent throughout the paper or potentially even separating these two goals into separate manuscripts.

2. A general question regarding the construction of Patterns of Interest: is the conceptualization of POIs with r = 1 appropriate, given that the stimuli used in “matched” conditions are still likely not identical? Is there a theoretical ceiling to correlation you could actually observe?

Additional:

Line 270: this seems to be a self-citation of an earlier version of this manuscript. Since it is presented as an introduction to the method used here, though, it seems circular and inappropriate. It makes it harder to understand what is seen as the main contribution of the present paper (and whether the cited paper is thought of as a separate work).

## Author Response

All author responses are indicated in blue

Text from manuscript text in 12pt, times new roman, indented

Reviewer 1

Enthusiasm is high for the topic of the study, that of processing of somatosensory (SS) stimulation, with an fMRI investigation of differential responses across the brain to hedonic, aversive and stimuli without a particular valence. This is an understudied field, and much of the cortical processing of hedonic vs. aversive SS stimuli is still poorly understood.

The enthusiasm for the topic is somewhat balanced (but not outweighed) by concerns about the data analyses. In particular, the approach used in the paper is a bit too sophisticated, consisting of RSA with mRDMs (it is not quite clear to me why the manuscript uses non-standard terms such as POI for mRDM -- this makes the manuscript more opaque than it needs to be for someone with knowledge of RSA), with a technique that aims to represent nRDMs through a weighted combination of mRDMs reflecting different neural tuning models (e.g., an mRDM reflecting neural representations coding for valence, for specific touch etc. etc.).

We thank the author for noting the similarity of our use of POI to previously described mRDM. Our choice of nomenclature was to reflect two distinctions between our approach and others of this family of approaches. Other approaches applying mRDM to observed representational data (nRDM), look for the best fit of individual models. In contrast, our approach involves a search for the combination of conceptual models that best approximates the nRDM. For this reason, we do not want to mislead readers and use of POI helps differentiate our approach, which (as outlined below), we believe allows more nuanced investigation of neural representation patterns than previously afforded.

That said, we also agree that it is important that the current paper give appropriate acknowledgment to its predecessors, and that it accurately reflects the similarity (and differences) between the current approach, and those conducted previously. See sections beginning at lines 103,

We generated combinations of predefined similarity matrices in an approach that is a variation of previously described applications of a priori conceptual models of representational dissimilarity matrices (mRDM; Kriegeskorte et al., 2008; Kriegeskorte and Kievit, 2013; Popal et al., 2019). We refer to our hypothesized similarity models as patterns of interest (POI) - each of which characterizes a correlation pattern that would be expected given perfect representation of a single vector of information, e.g., painful touch. Whereas previous approaches fit single hypothesized conceptual models to the observed data, the current POI approach (Kryklywy et al., 2021) looks for the simultaneous best fit of combinations of multiple models to observed representational patterns extracted from predefined regions of interest (ROIs).

Line 123

Critically, these analyses allow us to fit multiple overlapping pattern components simultaneously and identify the best fitting combination, rather than fitting each component alone and comparing the representational strength of each component in isolation.

and Line 280.

POIs are functionally similar to conceptual dissimilarity models (mRDM) for modeling content of RSA patterns (Kriegeskorte et al., 2008; Kriegeskorte and Kievit, 2013; Popal et al., 2019) in that they act to fit observed data to theory-based pre-specified representational patterns. Critically, however, the current approach is distinct from these in that it acts to fit combinations of conceptual patterns of interest to a given brain region, rather than individual models.

There are a couple of concerns with the technique: One is the sheer number of mRDMs -- there are 13 different mRDMs for different possibilities of neuronal tuning. Given that the mRDMs are not orthogonal, this creates the problem that there are multiple possible fits for a given nRDM. The authors introduce a greedy fitting strategy, which is sensible in terms of being computationally feasible, but the problem persists that there might be other fits than the ones shown that are either better or at least equally good but using different mRDM components.

This problem of arbitrariness/non-uniqueness of fits is exacerbated by the arbitrariness associated with the mRDMs themselves: For instance, in the “negative events” mRDM, aversive stimulation is weighed as much as the absence of an appetitive stimulation when expected. These are debatable, ad hoc choices, and it is unclear how different component weights here and in other matrices would change the results. These concerns about the rigor of the analyses reduces the study’s potential impact, and it would correspondingly strengthen the study if the authors could show that their findings are robust with respect to different choices in mRDM or fitting procedures.

At the least, the authors should address the arbitrariness of the mRDMs/POIs and the non-uniqueness of solutions and how this affects the strength of their conclusions in the Discussion.

Thank you for the opportunity to clarify our approach at its strengths and weaknesses. The decision to include a relatively large number of models was made specifically to address the multifunctional roles that many of the interrogated brain regions hold. Specifically, our method allows the researcher to assess the models in combination, and dissociate the portions of representational variance that can be attributed to different features. This approach enables us to do so, not in isolation, but in combination based on the assumption that a given brain region will be a weighted representation of multiple features.

POIS were based on evidence from affective science regarding how tactile and emotional information is processed, as well as knowledge of the variables manipulated in our own design. We acknowledge that these decisions are in part a priori decisions; designed and catered to the specific research questions we are targeting, and non-exhaustive of all potential POIs that could be used. We have now highlighted the factors in decision making in a section of the manuscript beginning line 289.

Note that the POIs were designed to capture information about the specific manipulations of the current experiment as well as cognitive processes associated with the ROIs defined in this study. The POIs we described have a particular focus on potential representations of tactile experience. Thus, they may not accurately reflect information represented in brain regions processing predominantly non-tactile information and are not intended to be an exhaustive model of all potential representational states.

We believe that our finding of robust results using multiple POIs highlights the potential of the technique in future applications. That said, we do believe that its implementation can move beyond this initial approach. These future applications, and the current limitations, are now outlined in the section beginning line 738.

It is notable that with the current implementation, all patterns of interest were defined as matrices of either 0 or 1 (i.e., perfect or no correlation). While the reliability of results we obtained (see the cross-validation procedures), and their consistency to hypothesized functioning in the tested regions (Craig, 2011; Cauda et al., 2012; Craig, 2015; Jin et al., 2015; Dixon et al., 2017; Miskovic and Anderson, 2018), support its implementation in this form, future work using similar technique may look to add additional refinement to these models. In many cases there may be a well-supported reason to expect partial, or scaled correlation between conditions, rather than absolutes. While the coarse approach to modeling implemented in the current work does provide meaningful insight into the nature of tactile representation, further refinement of the representational pattern investigated may provide additional details to this picture.

We do agree that there are potentially other representation pattern combinations as well as unexamined representational patterns that may explain the observed data at least as well as the identified results. This is certainly a limitation of the approach that we now specify in a limitations section beginning line 748.

An additional limitation to the current work that by constraining our search to a greedy best-first approach, we have by algorithmic definition limited the combinations of POIs tested. We believe that our current approach provides greater refinement towards understanding how multiple sources of information contribute in parallel to observed neural representational patterns than previous approaches and does so in a computationally efficient manner.

However, we believe that the search procedure implemented in the current work still allows greater refinement in understanding how multiple sources of information may be represented in a brain region when compared to the previous approaches fitting isolated vectors. That said, we do not believe our greedy best-first algorithm to be the only appropriate search technique, and hope this works encourages design, testing and comparison of other combination-fitting methods. This has been highlighted now as the discussion of limitations continues. See line 752.

That said, we also acknowledge the importance of future work to explore and compare alternate search algorithms as well as computationally intense brute-force approaches. We believe that POI-guided pattern component modeling represents an exciting new approach to multivariate analyses, and that its application to human neuroimaging data can allow for increasingly detailed ‘read out’ into how and where information in processed within the human brain.

There are some other concerns, in particular regarding the high rate of subjects whose data could not be processed: Out of 121 participants, preprocessing was not completed for 54 of them, including 12 for which data files were missing. These are somewhat high percentages and it would be good to better understand the reason for the large number of subjects for which preprocessing failed. Yet, the final dataset of 67 participants still seems sufficiently large.

This project was conducted across multiple centers, with most analyses conducted cross-continent (and across international borders) to the location of data collection. MEICA preprocessing are notoriously finicky, and these steps were performed remotely by the researcher on computational clusters local to the collection site, with the processed data then transfers to server local to the active research team. The result of this structure was that the temporary files generated during the preprocessing MECIA pipeline were inaccessible to the active researchers, and thus unable to be used in troubleshooting.

However, as the reviewer noted, we do believe that an n of 67, while disappointing given the collected sample size, is appropriate for the current analyses.

In summary, even with these concerns, given the understudied nature of SS processing mechanisms in the human brain and the study’s combination of valent and non-valent stimuli, with the changes suggested above, this study is an interesting addition to the literature!

We thank the reviewer for their input and insights and have worked diligently to address their concerns!

Minor:

l. 22: should be “emphasize"

fixed

l. 59 should be “differentiate"

fixed

l. 202: Lombardo et al. 2016 reference missing from reference list

added

l. 269: reference formatting messed up

fixed

l. 471: should be “represent"

fixed

-

Reviewer 2

The authors use a subset of a large-scale fMRI dataset to present a variation of pattern-component modeling for a-priori hypothesis testing which involves searching for “patterns of interest", or POIs, in various brain regions. The authors use this method to investigate representation of tactile information in the brain. Overall the subject of the manuscript is interesting and the manuscript is well written. My main concern for the manuscript is that it is difficult to figure out whether it is a methods paper or a research paper.

Main:

1. I am still confused about exactly how new the implementation of PCM used in this manuscript is, and how important the authors believe this aspect of the paper to be. On one hand, the authors state that they developed and applied it here, but there are also some citations that make it seem like it already exists. In general, if the focus of the paper were on the method, I would expect more validation and more introduction of the method in the manuscript. Instead, the method is introduced in the methods section only - and even there, the focus is more on the composition of the POIs than the implementation of the method. However, in the Conclusion and Abstract, the main contribution seems to be the development or extension of the method rather than the conclusions of the fMRI study analyzed. It would be helpful if the authors clarified their intentions by making the emphasis more consistent throughout the paper or potentially even separating these two goals into separate manuscripts.

The reviewers point about the confusion on the intention of the paper is well taken. The manuscript does indeed straddle the line between a methods and research article.

The intention was this work is to A) further investigate the cortical representations of hedonic vs. non-hedonic touch and B) highlight the novel methodologies developed while completing (A) in a manner that makes them accessible to other researcher for their own work. We believe that the greedy-best first pattern fitting analyses presented in the current work are a consequential extension of existing RSA techniques; however, we do not feel that it alone merits a methods paper, as it is the combination of two existing approaches: 1) conceptual model fitting to observed representational similarity patterns, and 2) greedy best-first search algorithms). In its current form, we believe that the work demonstrates the utility of the novel approach by generating meaningful insight into the representations of hedonic/non-hedonic touch, which is the primary contribution of the work.

We now indicate this hybrid intention in both the introduction and conclusion sections of the manuscript. See sections beginning line 25

Here we present one such approach, focusing on using a variant of pattern component modeling (PCM; Diedrichsen et al., 2011; Kriegeskorte and Kievit, 2013; Diedrichsen et al., 2018) that fits combinations of pre-specified conceptual similarity patterns to observed neural representational patterns to investigate instantiations of sensory and hedonic tactile information in the central nervous system (CNS).

line 38

Yet while discriminatory and hedonic information are clearly dissociable in the peripheral nervous system (PNS), investigating the dissociability vs integration of these signals as instantiated in the CNS remains a challenge, in part due to the limited analytic techniques for parcellating overlapping patterns of representation in the brain. The current work presents a novel approach to the analysis of human neuroimaging data that decomposes multivariate representational patterns (described via representational similarity analyses; RSA) into predefined representational patterns of interest (POIs) that model the information content instantiated by the BOLD response.

and 729 respectively

Analytic considerations

By iteratively fitting combinations of predefined models of representational patterns to observed data, rather than fitting each pattern in isolation, we were able to interrogate the representation of information with more conceptual resolution. Specifically, this approach allows researchers to determine whether individual brain regions represented information as either isolated or integrated patterns, as well as the strength of each representational pattern in the context of others. Applying this technique to targeted hypothesis-specific representations, we were able to identify meaningful differences in the cortical representation of discriminative versus hedonic somatosensory signals.

2. A general question regarding the construction of Patterns of Interest: is the conceptualization of POIs with r = 1 appropriate, given that the stimuli used in “matched” conditions are still likely not identical? Is there a theoretical ceiling to correlation you could actually observe?

As a first application of the analytic technique, we opted to go with the binary approach to representing our similarity of conditions. As a proof-of-concept, it was important to show that the technique could identify significant patterns in observed data, even when applied in a relatively coarse manner. That said, future implementation of the technique should work to create more refined/nuanced POIs, that could vary (based on theoretical expectation) in either the strength of expected between/within condition correlations, or even between subject, to assist with investigations of individual differences. We have added an additional section (beginning line 739) that highlights both these potential applications, as well as the current limitations.

Related to this, we have been working in parallel to this manuscript on adding functionality to the R package associated with this project that facilitates the implementation of these POI nuances: see Kryklywy JH, Forys BJ, Todd RM (2021) Pattern Component Modeling for R. In, 2.0 Edition, p R Package.

Section beginning Line 739:

Analytic considerations

By iteratively fitting combinations of predefined models of representational patterns to observed data, rather than fitting each pattern in isolation, we were able to interrogate the representation of information with more conceptual resolution. Specifically, this approach allows researchers to determine whether individual brain regions represented information as either isolated or integrated patterns, as well as the strength of each representational pattern in the context of others. Applying this technique to targeted hypothesis-specific representations, we were able to identify meaningful differences in the cortical representation of discriminative versus hedonic somatosensory signals.

It is notable that with the current implementation of POI-based PCM, all patterns of interest were defined as matrices of either 0 or 1 (i.e., perfect or no correlation). While the reliability of results we obtained (see the cross-validation procedures), and their consistency to hypothesized functioning in the tested regions (Craig, 2011; Cauda et al., 2012; Craig, 2015; Jin et al., 2015; Dixon et al., 2017; Miskovic and Anderson, 2018), support its implementation in this form, future work using similar technique may look to add additional refinement to these models. In many cases there may be a well-supported reason to expect partial, or scaled correlation between conditions, rather than absolutes. While the coarse approach to modeling implemented in the current work does provide meaningful insight into the nature of tactile representation, further refinement of the representational pattern investigated may provide additional details to this picture.

An additional limitation to the current work that by constraining our search to a greedy best-first approach, we have by algorithmic definition limited the combinations of POIs tested. We believe that our current approach provides greater refinement towards understanding how multiple sources of information contribute in parallel to observed neural representational patterns than previous approaches, and does so in a computationally efficient manner. That said, we also acknowledge the importance of future work to explore and compare alternate search algorithms as well as computationally intense brute-force approaches as the field further refines PCM analyses. We believe that POI-guided pattern component modeling represents an exciting new approach to multivariate analyses, and that its application to human neuroimaging data can allow for increasingly detailed ‘read out’ into how and where information in processed within the human brain.

Additional:

Line 270: this seems to be a self-citation of an earlier version of this manuscript. Since it is presented as an introduction to the method used here, though, it seems circular and inappropriate. It makes it harder to understand what is seen as the main contribution of the present paper (and whether the cited paper is thought of as a separate work).

The unintended citation here was to a previous version of the paper (a preprint at BioXriv) and has been removed.

## References

[B1] Abraham J, Mathew S (2019) Merkel cells: a collective review of current concepts. Int J Appl Basic Med Res 9:9–13. 10.4103/ijabmr.IJABMR_34_18 30820413 PMC6385537

[B2] Abraira VE, et al. (2017) The cellular and synaptic architecture of the mechanosensory dorsal horn. Cell 168:295–310.e19. 10.1016/j.cell.2016.12.010 28041852 PMC5236062

[B3] Anderson AK, Phelps EA (2002) Is the human amygdala critical for the subjective experience of emotion? Evidence of intact dispositional affect in patients with amygdala lesions. J Cogn Neurosci 14:709–720. 10.1162/08989290260138618 12167256

[B4] Arlot A, Celisse A (2010) A survey of cross-validation procedures. Statist Surv 4:40–79.

[B5] Baumgärtner U, Tiede W, Treede RD, Craig AD (2006) Laser-evoked potentials are graded and somatotopically organized anteroposteriorly in the operculoinsular cortex of anesthetized monkeys. J Neurophysiol 96:2802–2808. 10.1152/jn.00512.2006 16899640

[B6] Bushnell MC, Duncan GH, Hofbauer RK, Ha B, Chen JI, Carrier B (1999) Pain perception: is there a role for primary somatosensory cortex? Proc Natl Acad Sci U S A 96:7705–7709. 10.1073/pnas.96.14.7705 10393884 PMC33605

[B7] Cauda F, Costa T, Torta DM, Sacco K, D’Agata F, Duca S, Geminiani G, Fox PT, Vercelli A (2012) Meta-analytic clustering of the insular cortex: characterizing the meta-analytic connectivity of the insula when involved in active tasks. Neuroimage 62:343–355. 10.1016/j.neuroimage.2012.04.012 22521480 PMC4782788

[B8] Chikazoe J, Lee DH, Kriegeskorte N, Anderson AK (2014) Population coding of affect across stimuli, modalities and individuals. Nat Neurosci 17:1114–1122. 10.1038/nn.3749 24952643 PMC4317366

[B9] Chikazoe J, Lee DH, Kriegeskorte N, Anderson AK (2019) Distinct representations of basic taste qualities in human gustatory cortex. Nat Commun 10:1048. 10.1038/s41467-019-08857-z 30837463 PMC6401093

[B10] Corradi-Dell’Acqua C, Tusche A, Vuilleumier P, Singer T (2016) Cross-modal representations of first-hand and vicarious pain, disgust and fairness in insular and cingulate cortex. Nat Commun 7:10904. 10.1038/ncomms10904 26988654 PMC4802033

[B91] Cox RW (1996) AFNI: software for analysis and visualization of functional magnetic resonance neuroimages. Comput Biomed Res 29:162–173. 8812068 10.1006/cbmr.1996.0014

[B11] Craig AD (2011) Significance of the insula for the evolution of human awareness of feelings from the body. Ann N Y Acad Sci 1225:72–82. 10.1111/j.1749-6632.2011.05990.x 21534994

[B12] Craig AD (2015) How do you feel?: an interoceptive moment with your neurobiological self. Princeton: Princeton University Press.

[B13] Croy I, Luong A, Triscoli C, Hofmann E, Olausson H, Sailer U (2016) Interpersonal stroking touch is targeted to C tactile afferent activation. Behav Brain Res 297:37–40. 10.1016/j.bbr.2015.09.038 26433145

[B14] Diedrichsen J, Ridgway GR, Friston KJ, Wiestler T (2011) Comparing the similarity and spatial structure of neural representations: a pattern-component model. Neuroimage 55:1665–1678. 10.1016/j.neuroimage.2011.01.044 21256225 PMC3221047

[B15] Diedrichsen J, Yokoi A, Arbuckle SA (2018) Pattern component modeling: a flexible approach for understanding the representational structure of brain activity patterns. Neuroimage 180:119–133. 10.1016/j.neuroimage.2017.08.051 28843540

[B16] Dixon ML, Thiruchselvam R, Todd R, Christoff K (2017) Emotion and the prefrontal cortex: an integrative review. Psychol Bull 143:1033–1081. 10.1037/bul0000096 28616997

[B17] Dobs K, Isik L, Pantazis D, Kanwisher N (2019) How face perception unfolds over time. Nat Commun 10:1258. 10.1038/s41467-019-09239-1 30890707 PMC6425020

[B18] Doran JE, Michie D (1966) Experiments with the graph traverser program. Proc R Soc Lond A Math Phys Eng Sci 294:235–259.

[B19] Ehlers MR, Kryklywy JH, Beukers AO, Moore SR, Forys BJ, Anderson AK, Todd RM (2021) Reactivation of hedonic but not sensory representations in human emotional learning. bioRxiv. 10.1101/2021.11.25.469891.

[B20] Euston DR, Gruber AJ, McNaughton BL (2012) The role of medial prefrontal cortex in memory and decision making. Neuron 76:1057–1070. 10.1016/j.neuron.2012.12.002 23259943 PMC3562704

[B21] Everitt BJ, Cardinal RN, Parkinson JA, Robbins TW (2003) Appetitive behavior: impact of amygdala-dependent mechanisms of emotional learning. Ann N Y Acad Sci 985:233–250. 12724162

[B22] Fabozzi FJ (2014) The basics of financial econometrics: tools, concepts, and asset management applications. Hoboken: Wiley, Inc.

[B23] Gazzaniga MS, Ivry RB, Mangun GR (2019) Cognitive neuroscience: the biology of the mind, Ed 5. New York: W.W. Norton and Company.

[B24] Gazzola V, Spezio ML, Etzel JA, Castelli F, Adolphs R, Keysers C (2012) Primary somatosensory cortex discriminates affective significance in social touch. Proc Natl Acad Sci U S A 109:E1657–E1666. 10.1073/pnas.1113211109 22665808 PMC3382530

[B25] Giesecke T, Gracely RH, Grant MA, Nachemson A, Petzke F, Williams DA, Clauw DJ (2004) Evidence of augmented central pain processing in idiopathic chronic low back pain. Arthritis Rheum 50:613–623. 10.1002/art.20063 14872506

[B26] Goeleven E, De Raedt R, Leyman L, Verschuere B (2008) The Karolinska Directed Emotional Faces: a validation study. Cognition Emotion 22:1094–1118. 10.1080/02699930701626582

[B27] Hanke M, Halchenko YO, Sederberg PB, Hanson SJ, Haxby JV, Pollmann S (2009) PyMVPA: a python toolbox for multivariate pattern analysis of fMRI data. Neuroinformatics 7:37–53. 10.1007/s12021-008-9041-y 19184561 PMC2664559

[B28] Harjunen VJ, Spapé M, Ahmed I, Jacucci G, Ravaja N (2017) Individual differences in affective touch: behavioral inhibition and gender define how an interpersonal touch is perceived. Personality and Individual Differences 107:88–95. 10.1016/j.paid.2016.11.047

[B29] Haynes JD (2015) A primer on pattern-based approaches to fMRI: principles, pitfalls, and perspectives. Neuron 87:257–270. 10.1016/j.neuron.2015.05.025 26182413

[B30] Hiser J, Koenigs M (2018) The multifaceted role of the ventromedial prefrontal cortex in emotion, decision making, social cognition, and psychopathology. Biol Psychiatry 83:638–647. 10.1016/j.biopsych.2017.10.030 29275839 PMC5862740

[B31] Iggo A (1959) Cutaneous heat and cold receptors with slowly conducting (C) afferent fibres. Q J Exp Physiol Cogn Med Sci 44:362–370. 10.1113/expphysiol.1959.sp001417 13852621

[B32] Iggo A (1960) Cutaneous mechanoreceptors with afferent C fibres. J Physiol 152:337–353. 10.1113/jphysiol.1960.sp006491 13852622 PMC1363319

[B33] Jin J, Zelano C, Gottfried JA, Mohanty A (2015) Human amygdala represents the complete spectrum of subjective valence. J Neurosci 35:15145–15156. 10.1523/JNEUROSCI.2450-15.2015 26558785 PMC4642243

[B34] Kanwisher N, McDermott J, Chun MM (1997) The fusiform face area: a module in human extrastriate cortex specialized for face perception. J Neurosci 17:4302–4311. 10.1523/JNEUROSCI.17-11-04302.1997 9151747 PMC6573547

[B35] Knibestol M (1975) Stimulus-response functions of slowly adapting mechanoreceptors in the human glabrous skin area. J Physiol 245:63–80. 1127614 10.1113/jphysiol.1975.sp010835 PMC1330845

[B36] Kragel PA, Kano M, Van Oudenhove L, Ly HG, Dupont P, Rubio A, Delon-Martin C, Bonaz BL, Manuck SB, Gianaros PJ, Ceko M, Reynolds Losin EA, Woo CW, Nichols TE, Wager TD (2018) Generalizable representations of pain, cognitive control, and negative emotion in medial frontal cortex. Nat Neurosci 21:283–289. 10.1038/s41593-017-0051-7 29292378 PMC5801068

[B37] Kravitz DJ, Saleem KS, Baker CI, Ungerleider LG, Mishkin M (2013) The ventral visual pathway: an expanded neural framework for the processing of object quality. Trends Cogn Sci 17:26–49. 10.1016/j.tics.2012.10.011 23265839 PMC3532569

[B38] Kriegeskorte N, Kievit RA (2013) Representational geometry: integrating cognition, computation, and the brain. Trends Cogn Sci 17:401–412. 10.1016/j.tics.2013.06.007 23876494 PMC3730178

[B39] Kriegeskorte N, Mur M, Bandettini P (2008) Representational similarity analysis - connecting the branches of systems neuroscience. Front Syst Neurosci 2:4. 19104670 10.3389/neuro.06.004.2008 PMC2605405

[B40] Kryklywy JH, Ehlers MR, Anderson AK, Todd RM (2020) From architecture to evolution: multisensory evidence of decentralized emotion. Trends Cogn Sci 24:916–929. 32917534 10.1016/j.tics.2020.08.002

[B41] Kryklywy JH, Forys BJ, Todd RM (2021) Pattern component modeling for R. 1.0 edition. R Package.

[B42] Kundu P, Inati SJ, Evans JW, Luh WM, Bandettini PA (2012) Differentiating BOLD and non-BOLD signals in fMRI time series using multi-echo EPI. Neuroimage 60:1759–1770. 10.1016/j.neuroimage.2011.12.028 22209809 PMC3350785

[B43] Kundu P, Brenowitz ND, Voon V, Worbe Y, Vértes PE, Inati SJ, Saad ZS, Bandettini PA, Bullmore ET (2013) Integrated strategy for improving functional connectivity mapping using multiecho fMRI. Proc Natl Acad Sci U S A 110:16187–16192. 10.1073/pnas.1301725110 24038744 PMC3791700

[B44] Kundu P, Santin MD, Bandettini PA, Bullmore ET, Petiet A (2014) Differentiating BOLD and non-BOLD signals in fMRI time series from anesthetized rats using multi-echo EPI at 11.7 T. Neuroimage 102 [Pt 2]:861–874. 10.1016/j.neuroimage.2014.07.025 25064668

[B45] Kundu P, Voon V, Balchandani P, Lombardo MV, Poser BA, Bandettini PA (2017) Multi-echo fMRI: a review of applications in fMRI denoising and analysis of BOLD signals. Neuroimage 154:59–80. 10.1016/j.neuroimage.2017.03.033 28363836

[B46] Löken LS, Wessberg J, Morrison I, McGlone F, Olausson H (2009) Coding of pleasant touch by unmyelinated afferents in humans. Nat Neurosci 12:547–548. 10.1038/nn.2312 19363489

[B47] Lombardo MV, Auyeung B, Holt RJ, Waldman J, Ruigrok ANV, Mooney N, Bullmore ET, Baron-Cohen S, Kundu P (2016) Improving effect size estimation and statistical power with multi-echo fMRI and its impact on understanding the neural systems supporting mentalizing. Neuroimage 142:55–66. 10.1016/j.neuroimage.2016.07.022 27417345 PMC5102698

[B48] López-Solà M, Pujol J, Hernández-Ribas R, Harrison BJ, Ortiz H, Soriano-Mas C, Deus J, Menchón JM, Vallejo J, Cardoner N (2010) Dynamic assessment of the right lateral frontal cortex response to painful stimulation. Neuroimage 50:1177–1187. 10.1016/j.neuroimage.2010.01.031 20080188

[B90] Markello RD, Spreng RN, Luh W-M, Anderson AK, De Rosa E (2018) Segregation of the human basal forebrain using resting state functional MRI. NeuroImage 173:287–297. 29496614 10.1016/j.neuroimage.2018.02.042

[B49] Marshall AG, McGlone FP (2020) Affective touch: the enigmatic spinal pathway of the C-tactile afferent. Neurosci Insights 15:2633105520925072. 10.1177/2633105520925072 32529186 PMC7265072

[B50] Marshall AG, Sharma ML, Marley K, Olausson H, McGlone FP (2019) Spinal signalling of C-fiber mediated pleasant touch in humans. Elife 8:e51642. 10.7554/eLife.51642 31872799 PMC6964968

[B51] McBurney DH, Balaban CD (2009) A heuristic model of sensory adaptation. Atten Percept Psychophys 71:1941–1961. 10.3758/APP.71.8.1941 19933575

[B52] McFarland DJ, Sibly RM (1975) The behavioural final common path. Philos Trans R Soc Lond B Biol Sci 270:265–293. 10.1098/rstb.1975.0009 239416

[B53] McGlone F, Reilly D (2010) The cutaneous sensory system. Neurosci Biobehav Rev 34:148–159. 10.1016/j.neubiorev.2009.08.004 19712693

[B54] McGlone F, Wessberg J, Olausson H (2014) Discriminative and affective touch: sensing and feeling. Neuron 82:737–755. 10.1016/j.neuron.2014.05.001 24853935

[B55] Miskovic V, Anderson AK (2018) Modality general and modality specific coding of hedonic valence. Curr Opin Behav Sci 19:91–97. 10.1016/j.cobeha.2017.12.012 29967806 PMC6024250

[B56] Moore SR (2017) Sources of individual variability in sensitivity to the environment. PhD thesis, Cornell University.

[B57] Morris JS, Ohman A, Dolan RJ (1998) Conscious and unconscious emotional learning in the human amygdala. Nature 393:467–470. 10.1038/30976 9624001

[B58] Morrison I (2016) Keep calm and cuddle on: social touch as a stress buffer. Adapt Human Behav Physiol 2:344–362. 10.1007/s40750-016-0052-x

[B59] Mur M, Bandettini PA, Kriegeskorte N (2009) Revealing representational content with pattern-information fMRI–an introductory guide. Soc Cogn Affect Neurosci 4:101–109. 10.1093/scan/nsn044 19151374 PMC2656880

[B60] Nagi SS, Marshall AG, Makdani A, Jarocka E, Liljencrantz J, Ridderström M, Shaikh S, O’Neill F, Saade D, Donkervoort S, Foley AR, Minde J, Trulsson M, Cole J, Bönnemann CG, Chesler AT, Bushnell MC, McGlone F, Olausson H (2019) An ultrafast system for signaling mechanical pain in human skin. Sci Adv 5:eaaw1297. 10.1126/sciadv.aaw1297 31281886 PMC6609212

[B61] Neubarth NL, Emanuel AJ, Liu Y, Springel MW, Handler A, Zhang Q, Lehnert BP, Guo C, Orefice LL, Abdelaziz A, DeLisle MM, Iskols M, Rhyins J, Kim SJ, Cattel SJ, Regehr W, Harvey CD, Drugowitsch J, Ginty DD (2020) Meissner corpuscles and their spatially intermingled afferents underlie gentle touch perception. Science 368:eabb2751. 10.1126/science.abb2751 32554568 PMC7354383

[B62] Nielsen CS, Staud R, Price DD (2009) Individual differences in pain sensitivity: measurement, causation, and consequences. J Pain 10:231–237. 10.1016/j.jpain.2008.09.010 19185545

[B63] Nieuwenhuys R (2012) The insular cortex: a review. Prog Brain Res 195:123–163. 10.1016/B978-0-444-53860-4.00007-6 22230626

[B64] Olausson H, Lamarre Y, Backlund H, Morin C, Wallin BG, Starck G, Ekholm S, Strigo I, Worsley K, Vallbo AB, Bushnell MC (2002) Unmyelinated tactile afferents signal touch and project to insular cortex. Nat Neurosci 5:900–904. 10.1038/nn896 12145636

[B65] Orenius TI, Raij TT, Nuortimo A, Näätänen P, Lipsanen J, Karlsson H (2017) The interaction of emotion and pain in the insula and secondary somatosensory cortex. Neuroscience 349:185–194. 10.1016/j.neuroscience.2017.02.047 28259800

[B66] Pessoa L, Adolphs R (2010) Emotion processing and the amygdala: from a ‘low road’ to ‘many roads’ of evaluating biological significance. Nat Rev Neurosci 11:773–783. 10.1038/nrn2920 20959860 PMC3025529

[B67] Picard RR, Cook RD (1984) Cross-validation of regression models. J Am Stat Assoc 79:575–583. 10.1080/01621459.1984.10478083

[B68] Pinel JPJ, Barnes S (2018) Biopsychology. Ed 10. Hoboken: Pearson Higher Education.

[B69] Pollatos O, Herbert BM, Mai S, Kammer T (2016) Changes in interoceptive processes following brain stimulation. Philos Trans R Soc Lond B Biol Sci 371:20160016. 28080973 10.1098/rstb.2016.0016 PMC5062104

[B70] Popal H, Wang Y, Olson IR (2019) A guide to representational similarity analysis for social neuroscience. Soc Cogn Affect Neurosci 14:1243–1253. 10.1093/scan/nsz099 31989169 PMC7057283

[B71] Posse S, Wiese S, Gembris D, Mathiak K, Kessler C, Grosse-Ruyken ML, Elghahwagi B, Richards T, Dager SR, Kiselev VG (1999) Enhancement of BOLD-contrast sensitivity by single-shot multi-echo functional MR imaging. Magn Reson Med 42:87–97. 10.1002/(SICI)1522-2594(199907)42:1<87::AID-MRM13>3.0.CO;2-O 10398954

[B72] Qiu YH, Noguchi Y, Honda M, Nakata H, Tamura Y, Tanaka S, Sadato N, Wang XH, Inui K, Kakigi R (2006) Brain processing of the signals ascending through unmyelinated C fibers in humans: an event-related functional magnetic resonance imaging study. Cereb Cortex 16:1289–1295. 10.1093/cercor/bhj071 16280463

[B73] Rolls ET (2000) Précis of The brain and emotion. Behav Brain Sci 23:177–191; discussion 192–233. 10.1017/s0140525x00002429 11301577

[B74] Rolls ET (2019) Emotion and reasoning in human decision-making. Economics 13:2019–2039. 10.5018/economics-ejournal.ja.2019-39

[B75] Rolls ET, O’Doherty J, Kringelbach ML, Francis S, Bowtell R, McGlone F (2003) Representations of pleasant and painful touch in the human orbitofrontal and cingulate cortices. Cereb Cortex 13:308–317. 10.1093/cercor/13.3.308 12571120

[B76] Strigo IA, Craig AD (2016) Interoception, homeostatic emotions and sympathovagal balance. Philos Trans R Soc Lond B Biol Sci 371:20160010. 28080968 10.1098/rstb.2016.0010 PMC5062099

[B77] Todd RM, Cunningham WA, Anderson AK, Thompson E (2012) Affect-biased attention as emotion regulation. Trends Cogn Sci 16:365–372. 10.1016/j.tics.2012.06.003 22717469

[B78] Todd RM, Miskovic V, Chikazoe J, Anderson AK (2020) Emotional objectivity: neural representations of emotions and their interaction with cognition. Annu Rev Psychol 71:25–48. 10.1146/annurev-psych-010419-051044 31610131

[B79] Vallbo AB, Olausson H, Wessberg J (1999) Unmyelinated afferents constitute a second system coding tactile stimuli of the human hairy skin. J Neurophysiol 81:2753–2763. 10.1152/jn.1999.81.6.2753 10368395

[B80] Vierck CJ, Whitsel BL, Favorov OV, Brown AW, Tommerdahl M (2013) Role of primary somatosensory cortex in the coding of pain. Pain 154:334–344. 10.1016/j.pain.2012.10.021 23245864 PMC4501501

[B81] Visser RM, Scholte HS, Beemsterboer T, Kindt M (2013) Neural pattern similarity predicts long-term fear memory. Nat Neurosci 16:388–390. 10.1038/nn.3345 23434912

[B82] Visser RM, Kunze AE, Westhoff B, Scholte HS, Kindt M (2015) Representational similarity analysis offers a preview of the noradrenergic modulation of long-term fear memory at the time of encoding. Psychoneuroendocrinology 55:8–20. 10.1016/j.psyneuen.2015.01.021 25705798

[B83] Visser RM, de Haan MI, Beemsterboer T, Haver P, Kindt M, Scholte HS (2016) Quantifying learning-dependent changes in the brain: single-trial multivoxel pattern analysis requires slow event-related fMRI. Psychophysiology 53:1117–1127. 10.1111/psyp.12665 27153295

[B84] Vuilleumier P (2005) How brains beware: neural mechanisms of emotional attention. Trends Cogn Sci 9:585–594. 10.1016/j.tics.2005.10.011 16289871

[B85] Whitsel BL, Favorov OV, Li Y, Quibrera M, Tommerdahl M (2009) Area 3a neuron response to skin nociceptor afferent drive. Cereb Cortex 19:349–366. 10.1093/cercor/bhn086 18534992 PMC2638786

